# Total synthesis of the O-antigen repeating unit of *Providencia stuartii* O49 serotype through linear and one-pot assemblies

**DOI:** 10.3762/bjoc.17.199

**Published:** 2021-12-13

**Authors:** Tanmoy Halder, Somnath Yadav

**Affiliations:** 1 Department of Chemistry, Indian Institute of Technology (ISM), Dhanbad, 826004, Jharkhand, India

**Keywords:** capsular polysaccharide, carbohydrate vaccines, O-antigen, oligosaccharide synthesis, one-pot synthesis

## Abstract

Capsular polysaccharides of pathogenic bacteria have been reported to be effective vaccines against diseases caused by them. *Providencia stuartii* is a class of enterobacteria of the family Providencia that is responsible for several antibiotic resistant infections, particularly urinary tract infections of patients with prolonged catheterization in hospital settings. Towards the goal of development of vaccine candidates against this pathogen, we herein report the total synthesis of a trisaccharide repeating unit of the O-antigen polysaccharide of the *P. stuartii* O49 serotype containing the →6)-β-ᴅ-Gal*p*-(1→3)-β-ᴅ-Gal*p*NAc(1→4)-α-ᴅ-Gal*p*(1→ linkage. The synthesis of the trisaccharide repeating unit was carried out first by a linear strategy involving the [1 + (1 + 1 = 2)] assembly, followed by a one-pot synthesis involving [1 + 1 + 1] strategy from the corresponding monosaccharides. The one-pot method provided a higher yield of the protected trisaccharide intermediate (73%) compared to the two step synthesis (66%). The protected trisaccharide was then deprotected and *N*-acetylated to finally afford the desired trisaccharide repeating unit as its α-*p*-methoxyphenyl glycoside.

## Introduction

O-antigens or O-specific polysaccharides are one of the important constituents of the surface lipopolysaccharides (LPS) of the cell wall of Gram-negative bacteria. These antigens are responsible for several functions that include adhesion to the host cells and are also found to contribute to the evasion of the host immune responses. Structurally, the O-antigens consist of polysaccharide repeating units bearing several different monosaccharides. Due to their importance in regulating the host’s immune system, the bacterial cell surface LPS in general and the O-antigens in particular have been proposed and reported as candidates for vaccine development [1–8]. This objective has been proposed to be achieved by the synthesis of chemically homogeneous glycoconjugates bearing the O-antigen oligosaccharide conjugated to peptides for eliciting the desired immune response through vaccines [9–21]. For the above purposes, large scale access to pure, defined, and homogeneous samples of the desired LPS oligosaccharides are essential for realization of the goal towards vaccine development against these pathogens [1–21].

*Providencia* is a genus of Gram-negative bacteria that belongs to the enterobacteria family and is responsible for causing several enteric infections including urinary tract infections. *Providencia* includes mainly five virulent species – *P. alcalifaciens*, *P. rettgeri*, *P. rustigianii*, *P. stuartii*, and *P. heimbachae* [22]. The *Providencia* species has been isolated from urine, stool, blood, throat, axilla, and perineum of infected patients as also from polluted soil and wastewater [22–24]. Of the five species, the clinically important ones are *Providencia rettgeri* and *P. stuartii* which are found to be particularly responsible for antibiotic resistant infections in hospitalized patients with long term urinary catheters, particularly immuno-compromised patients [25]. A total 61% of urinary region specimens in the infected populace consist of either *Providencia stuartii* or *Proteus mirabilis*, and the infections may even lead to fatal bacteremia [22,25].

**Figure 1:**
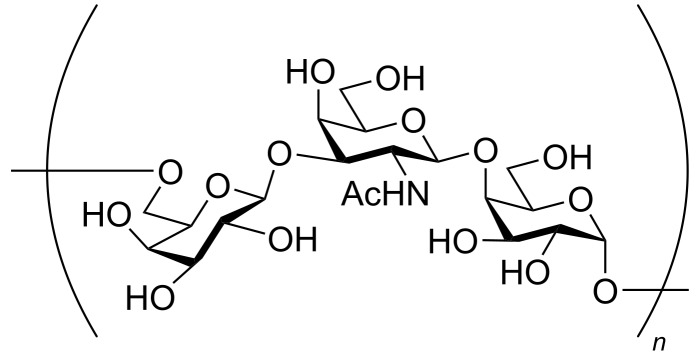
Structure of the repeating unit of the lipopolysaccharide of *Providencia stuartii* O49 serotype.

The structure of repeating oligosaccharide units of *O*-polysaccharides of several *Providencia* O-serogroups as well as *P. stuartii* O4 [26], O18 [27], O20 [28] O33 [29], O43 [30], O44 [31], O47 [32], and O57 [33] have been reported. With respect to the synthesis of the O-antigens, that of *Providencia rustigianii* O34 was reported by Mukhopadhyay et al. in 2013 [34]. Chheda and co-worker, in 2015, reported the total synthesis of the pentasaccharide repeating unit of the *O*-specific polysaccharide of *Providencia alcalifaciens* O28 [35]. In 2017, Kulkarni and co-worker accomplished the total synthesis of a O-polysaccharide of *Providencia alcalifaciens* O22 via a one pot assembly of the oligosaccharide unit [36]. Recently in 2020 Werz and co-workers completed the total synthesis of a tri-, hexa-, and heptasaccharide of the O-polysaccharide of *Providencia rustigianii* O34 [37]. In the context of the O-antigen repeating units of various *P. stuartii* serotypes, in 2004 Bushmarinov et al. [38] reported the O-antigen of the O49 serotype as consisting of the →6)-β-ᴅ-Gal*p*-(1→3)-β-ᴅ-Gal*p*NAc(1→4)-α-ᴅ-Gal*p*(1→ linkage (Figure 1). Herein, we report the total synthesis of the hitherto not synthesized above trisaccharide repeating unit containing two ᴅ-galactose and one *N*-acetyl-ᴅ-galactosamine moieties. The linear synthesis of the target oligosaccharide was first carried out via a linear [1 + (1 + 1 = 2)] assembly of a galactopyranose donor with a Gal*p*NTroc–Gal*p* acceptor. We also demonstrated a follow-up one-pot synthesis involving a [1 + 1 + 1] strategy using the corresponding appropriately protected monosaccharides, providing the opportunity for rapid access to the desired target molecule.

## Results and Discussion

**Scheme 1:**
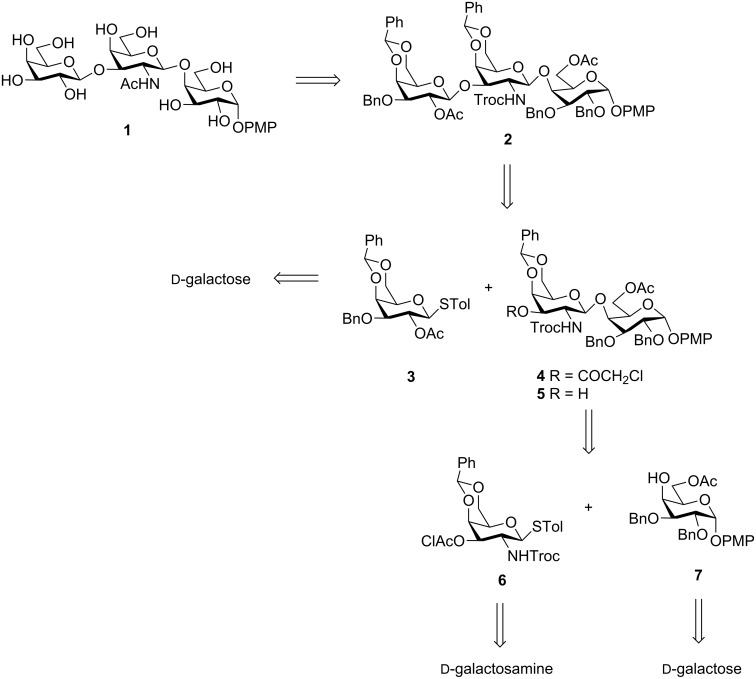
Retrosynthetic analysis for the synthesis of the target trisaccharide **1**.

The retrosynthesis of the target trisaccharide **1**, led to the monosaccharides **3**, **6**, and **7** (Scheme 1). The choice of the *p*-methoxyphenyl (PMP) group at the reducing end was based on the fact that it could be easily synthesized stereoselectively to mimic the α-glycosidic linkage in the native oligosaccharide. Further, the trisaccharide could be adapted for further conjugation to other moieties towards the synthesis of vaccine conjugates by removal of the anomeric PMP group. This has been previously demonstrated by removal of the anomeric PMP group, conversion to a glycosidic donor, and further conjugation to either linkers or amino acids by several authors [39–42]. The starting materials **3**, **6**, and **7** were also amenable to a [1 + 1 + 1] one-pot synthetic strategy by adopting minor synthetic modifications.

**Scheme 2:**
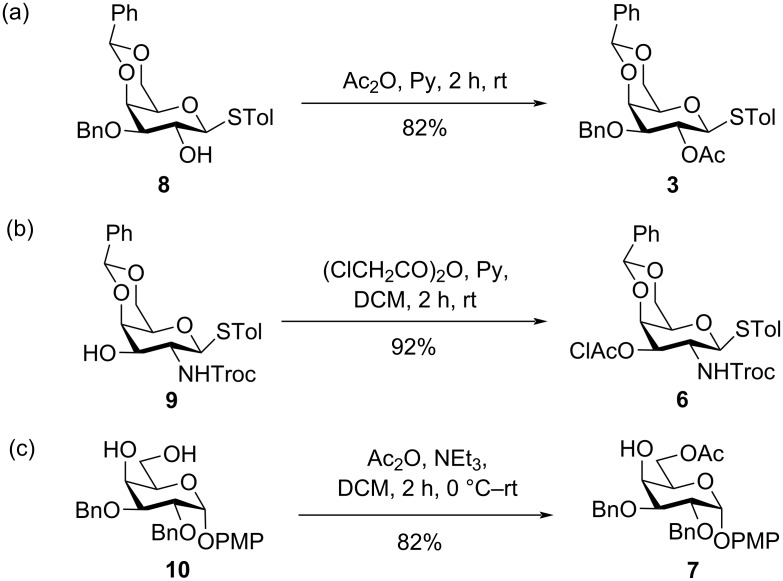
Synthesis of the monosaccharide building blocks **3**, **6**, and **7**.

The monosaccharide building blocks **3**, **6**, and **7** [43] were synthesized from previously reported compounds **8** [36], **9** [44–45], and **10** [46] as described in Scheme 2.

**Scheme 3:**
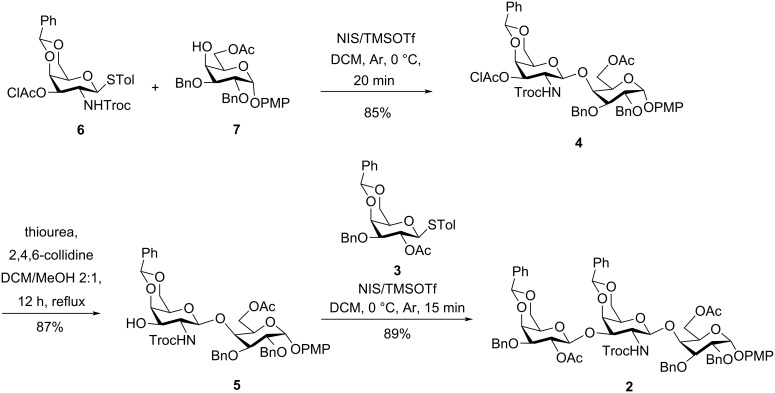
Linear synthesis of trisaccharide derivative **2**.

With the monosaccharide building blocks in hand, the galactosamine donor **6** was coupled with galactose acceptor **7** by activation of the thioglycoside using *N*-iodosuccinimide (NIS) in the presence of TMSOTf to afford the desired disaccharide β-ᴅ-Gal*p*NHTroc-(1→4)-α-ᴅ-Gal*p* (**4**) in 85% yield, as a single isomer (Scheme 3). Then, the chloroacetyl group was selectively removed using thiourea and 2,4,6-collidine [47] to afford the 3’-OH acceptor **5** in 87% yield. Finally, NIS/TMSOTf-promoted coupling of donor **3** with disaccharide acceptor **5** provided the desired β-linked trisaccharide **2** in 89% yield (Scheme 3).

**Scheme 4:**
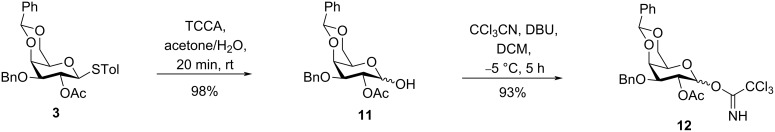
Synthesis of ᴅ-galactose donor **12**.

The linear synthesis of oligosaccharides is associated with several disadvantages such as multiple steps involving multiple work-up procedures, purifications requiring long time, manpower and the resulting high cost of synthesis and production. Consequently one-pot strategies have recently attracted a lot of attention and several methodologies have been developed as a potential solution that can reduce cost via bringing down solvent and time consumption [48–50]. In view of this, we next attempted the one-pot synthesis of the trisaccharide derivative **2** via a [1 + 1 + 1] approach. Initial studies using the thioglycoside donor **3** were not very fruitful, affording a complex mixture of products. Therefore, we modified our strategy to include its trichloroacetimidate derivative as the donor. The thioglycoside **3** was converted to the anomeric hydroxide using trichloroisocyanuric acid (TCCA) in aqueous acetone [51] resulting in compound **11**. Treatment of compound **11** with trichloroacetonitrile and DBU in dry DCM resulted in the formation of the desired trichloroacetimidate donor **12** in 93% yield (Scheme 4).

**Scheme 5:**
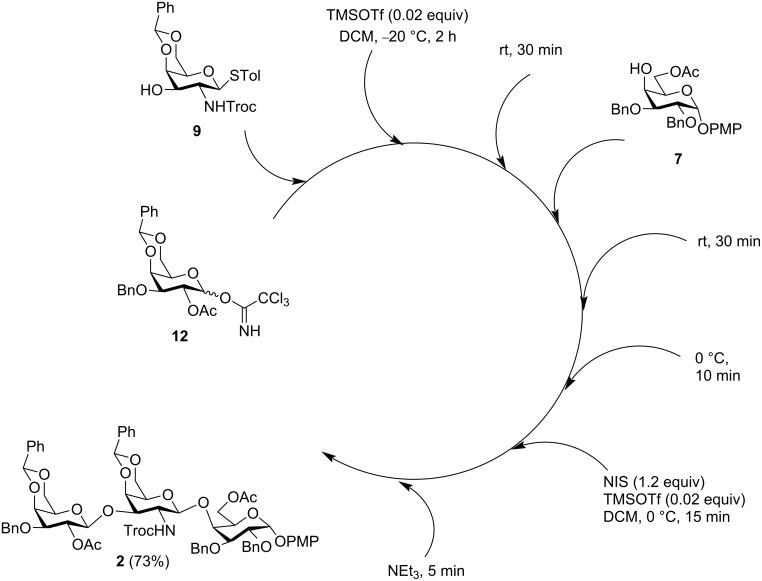
One-pot synthesis of trisaccharide derivative **2**.

The first stage of the one-pot synthesis was carried out using donor **12** and acceptor **9** in the presence of TMSOTf as promoter (Scheme 5). After 1 h of reaction, TLC monitoring indicated the full consumption of the starting materials. Analysis of a small aliquot of the reaction mixture by HRMS confirmed the formation of the desired disaccharide. Then, to the same pot, the second monosaccharide acceptor **7** was added, followed by the addition of NIS and TMSOTf. After 15 min of reaction, TLC monitoring showed complete consumption of the donor. Work-up of the reaction mixture followed by chromatographic purification afforded the pure trisaccharide **2** as a single isomer in an overall yield of 73%. The structure of the trisaccharide was confirmed by comparison of its NMR and HRMS spectral data with that of the previously synthesized product by the linear strategy.

**Scheme 6:**
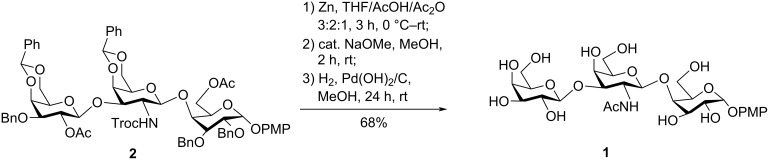
Synthesis of trisaccharide derivative **1**.

With the protected trisaccharide **2** in hand, it remained to carry out the *N*-acetylation and the removal of the protecting groups on the hydroxy groups. First, the concomitant removal of the Troc group and the *N*-acetylation was achieved using Zn/AcOH/Ac_2_O 3:2:1 as reagent in one pot (Scheme 6) [52]. Then, *O*-deacetylation was accomplished by using a catalytic amount of NaOMe in MeOH at room temperature. Finally, the benzylidene and the benzyl groups were removed by hydrogenolysis using 10% Pd(OH)_2_/C in methanol at ambient temperature with a H_2_ balloon, which afforded the target trisaccharide **1** in 68% yield over three steps (Scheme 6). The structure of **1** was confirmed by several NMR spectroscopic techniques such as ^1^H NMR, DEPT-135, ^13^C NMR, COSY, and HSQC as well as mass spectrometry using HRMS. The NMR data were found to correlate well with the data reported for the natural polysaccharide [38].

## Conclusion

In conclusion, the trisaccharide repeating unit of the O-polysaccharide of *Providencia stuartii* O49 in its *p*-methoxyphenyl glycoside form **1** was synthesized through a linear [1 + (1 + 1 = 2)] strategy. The target trisaccharide was synthesized as its *p*-methoxyphenyl glycoside that offered the unaltered stereochemistry of the sugar at the reducing end to mimic the glycosidic linkage of the natural polysaccharide. The target protected trisaccharide was also synthesized through a [1 + 1 + 1] one-pot strategy involving sequential glycosylations from the reducing end to the non-reducing end. The one-pot synthesis provided the final trisaccharide in an overall yield of 73% compared to the overall yield of 66% from the two step synthesis, though the former involved two extra steps for the synthesis of the first glycosidic donor and one chromatographic separation. The synthesis of the desired product was achieved through manipulations of the appropriate protecting group on the monosaccharides and subsequent realization of stereoselective glycosylations. The work provides an access to the trisaccharide repeating unit of the *O*-polysaccharide of *Providencia stuartii* O49 with the stereospecific α-*p*-methoxyphenyl glycoside.

## Supporting Information

File 1 Detailed experimental procedures and synthesis of compounds.

File 2 Copies of ^1^H and ^13^C NMR spectra of all known and new compounds synthesized.
