# Supplementary material for: Total synthesis of the O-antigen repeating unit of Providencia stuartii O49 serotype through linear and one-pot assemblies
Source: Beilstein J Org Chem. 2021 Dec 13;17:2915–21. doi: 10.3762/bjoc.17.199 (PMC8685571; doi:10.3762/bjoc.17.199)
Supplement: File 1 — Detailed experimental procedures and synthesis of compounds. [file Beilstein_J_Org_Chem-17-2915-s001.pdf]

BEILSTEIN JOURNAL OF ORGANIC CHEMISTRY

Supporting Information

for

Total synthesis of the O-antigen repeating unit of *Providencia stuartii* O49 serotype through linear and one-pot assemblies

Tanmoy Halder and Somnath Yadav

Beilstein J. Org. Chem. **2021,** *17,* 2915–2921. doi:10.3762/bjoc.17.199

Detailed experimental procedures and synthesis of compounds

License and Terms: This is a supporting information file under the terms of the Creative Commons Attribution License (https://creativecommons.org/licenses/by/4.0). Please note that the reuse, redistribution and reproduction in particular requires that the author(s) and source are credited and that individual graphics may be subject to special legal provisions.
The license is subject to the *Beilstein Journal of Organic Chemistry* terms and conditions: (https://www.beilstein-journals.org/bjoc/terms)

Experimental

General methods. All starting materials chemicals and reagents were purchased from commercial sources and were used without further purification. Anhydrous solvents, when required, were prepared according to standard procedures [1]. Reactions were carried out in inert atmosphere maintained either with nitrogen or argon. Column chromatography was performed using silica gel (230–400 mesh) under medium pressure. Petroleum ether (PE) used of the boiling range 60–80 °C. TLC was performed on precoated aluminum plates of silica gel 60-F_{254}. TLC spots were visualized by staining with vanillin solution and subsequent heating on a hot plate. All new compounds were characterized using ^{1}H, ^{13}C, DEPT and 2D-NMR spectroscopy, IR spectroscopy, HRMS, and specific rotation. Known compounds were characterized using ^{1}H and ^{13}C NMR spectroscopy, IR spectroscopy, HRMS, and specific rotation. Melting points were determined using open capillary method and are reported uncorrected. ^{1}H NMR spectra were recorded on a 400 MHz NMR spectrometer as solutions in $CDCl_3$ or D_2O. ^{13}C NMR spectra were recorded at 100 MHz. Chemical shifts δ are reported in parts per million (ppm) and are referenced to the residual $CHCl_3$ peak at δ = 7.26 for ^{1}H NMR and δ = 77.16 for ^{13}C NMR spectra. Coupling constants (*J*) are reported in hertz. Optical rotations were measured using a digital polarimeter. HRMS were recorded on a QTOF-Micro spectrometer. Compounds **8** [2,3], **9** [4], and **10** [5] are known compounds.

***p*-Tolyl 2-*O*-acetyl-3-*O*-benzyl-4,6-*O*-benzylidene-1-thio-β-D-galacto-pyranoside (3).** To a solution of **3** (0.390 g, 0.83 mmol) and pyridine (0.074 mL, 0.92 mmol), acetic anhydride (0.087 mL, 0.92 mmol) was added and the solution was stirred at room temperature for 2 h under a nitrogen atmosphere. After completion of

the reaction (as confirmed by TLC 30% EA/PE), the reaction mixture was diluted with EA and the organic layer was washed with water. The organic layer was separated and the aqueous part was extracted thrice with EA. The combined organic layer was washed with brine, dried over anhydrous Na_2SO_4, filtered, and then concentrated *in vacuo*. The crude product was purified by column chromatography using 25% EA/PE to yield **3** as a white solid (0.350 g, 82%): M. p. 125-127 °C. $[\alpha]_D^{25}$ = -27.6 (*c* = 0.13, $CHCl_3$); IR (cm^{-1}, $CHCl_3$) 2869, 1750, 1738, 1495, 1367, 1235, 1099, 727, 697. ^1H NMR (400 MHz, $CDCl_3$): δ 7.50 (d, *J* = 8 Hz, 2H, Ar*H*), 7.42-7.40 (m, 2H, Ar*H*), 7.36-7.28 (m, 8H, Ar*H*), 7.06 (d, *J* = 8 Hz, 2H, Ar*H*), 5.43 (s, 1H, PhC*H*), 5.26 (t, *J* = 9.7 Hz, 1H, H-2), 4.66 (d, *J* = 12.6 Hz, 1H, Bn*H*), 4.58 (d, *J* = 12.6 Hz, 1H, Bn*H*), 4.57 (d, *J* = 9.8 Hz, 1H, H-1), 4.34 (dd, *J* = 12.2 and 1.3 Hz, 1H, H-6), 4.17 (d, *J* = 3.2 Hz, 1H, H-4), 3.98 (dd, *J* = 12.4 and 1.5 Hz, 1H, H-6), 3.60 (dd, *J* = 9.6 and 3.4 Hz, 1H, H-3), 3.42 (s, 1H H-5), 2.33 (s, 3H, CH_3), 2.09 (s, 3H, COCH_3). ^{13}C NMR (100 MHz, $CDCl_3$): δ 169.3, 138.3, 138.0, 137.7, 134.3, 129.6, 129.2, 128.5, 128.2, 127.9, 127.7, 127.6, 126.8, 101.4 (Ph*C*H), 85.4 (C-1), 78.6 (C-3), 73.2 (C-4), 71.2, (Ph*C*H_2), 70.0 (C-5), 69.4 (C-6), 68.4(C-2), 21.4, 21.2. HRMS (ESI-TOF): calcd for $C_{29}H_{30}O_6SNa^+$ (M + Na) $^+$, 529.1655; found, 529.1651.

***p*-Tolyl 4,6-*O*-benzylidene-3-*O*-chloroacetyl-2-(2,2,2-trichloroethoxy-carbonylamino)-2-deoxy-1-thio-β-D-galactopyranoside (6).** To a solution of **13** (0.116 g, 0.21 mmol) in DCM (1 mL) at 0 °C, chloroacetic anhydride (0.073 g , 0.43 mmol) and pyridine (0.069 mL, 0.86 mmol) were added. Then, the solution was stirred at room temperature for 2 h whend TLC (50% EA/PE) showed complete conversion of the starting material. The solvent was evaporated *in vacuo* and the mixture was co-evaporated with toluene to remove residual pyridine. The sticky residue was purified by silica-gel column chromatography using 35% EA/PE as the eluent to

afford **6** (0.121 g, 92%) as a white solid. M. p. 125-127 °C·. $[\alpha]_D^{25}$ = -10.0 (c = 0.16, $CHCl_3$); IR (cm^{-1}, $CHCl_3$) 1754, 1712, 1634, 1494, 1408, 1451, 1371, 1273, 1168, 651, 597. ^1H NMR (400 MHz, $CDCl_3$): δ 7.55 (d, *J* = 8.1 Hz, 2H. Ar*H*), 7.42-7.34 (m, 4H, Ar*H*), 7.08 (d, *J* = 7.8 Hz, 2H, Ar*H*), 5.49 (s, 1H, PhC*H*), 5.44 (dd, *J* = 10.7, 3.0 Hz, 1H, H-3), 5.17 (d, *J* = 8.2 Hz, 1H, -N*H*), 5.06 (d, *J* = 10.0 Hz, 1H, H-1), 4.73 (m, 2H, CH_2CCl_3), 4.40-4.37 (m, 2H, H-6, H-4,), 4.08-3.98 (m, 3H, H-5, $OCOCH_2Cl$), 3.85-3.78 (m, 1H, H-2), 3.64 (bs, 1H, H-5), 2.34 (s, 3H, CH_3) ^{13}C NMR (100 MHz, $CDCl_3$): δ 167.3, 153.7, 138.7, 137.6, 134.4, 129.9, 129.4, 128.3, 127.0, 126.6, 101.1, 95.5, 84.6 (C-1), 74.5 (CH_2 of NHTroc), 73.1 (C-3, C-4), 69.6 (C-5), 69.3 (C-6), 50.4 (C-2), 40.9 (OCH_2Cl), 21.4 HRMS (ESI-TOF): calcd for $C_{24}H_{25}Cl_4NO_6SNa^+$ $(M + Na)^+$, 645.9998; found, 645.9999.

4-Methoxyphenyl 6-*O*-acetyl-2,3-di-*O*-benzyl-α-D-galactopyranoside (7). To a solution of **17** (0.200 g, 0.42 mmol) in DCM (2 mL), triethylamine (0.53 mL, 3.85 mmol) and acetic anhydride (0.044 mL, 0.47 mmol) were added. The reaction mixture was stirred at 0 °C to room temperature for 2 h, when TLC (60% EA/PET) showed complete conversion of the starting material. The reaction mixture was concentrated *in vacuo* and diluted with ethyl acetate. The organic layer was washed with water. The organic layer was separated and the aqueous part was extracted thrice with EA. The combined organic layer was washed with brine, dried over anhydrous Na_2SO_4, filtered, and concentrated *in vacuo*. The crude product was purified by column chromatography using 25% EA/PE as eluent to afford **7** (0.178 g, 82%) as a colorless sticky liquid. $[\alpha]_D^{25}$ = 101.7 (*c* = 0.5, $CHCl_3$); IR (cm^{-1}, $CHCl_3$) 3461, 1743, 1712, 1507, 1453, 1373, 1274, 1214, 1138, 1100, 1036, 738, 697 ^1H NMR (400 MHz, $CDCl_3$): δ 7.42-7.27 (m, 10H, Ar*H*), 7.05-7.01 (m, 2H, Ar*H*), 6.85-6.80 (m, 2H, Ar*H*), 5.36 (d, *J* = 3.6 Hz, 1H, H-1), 4.88 (d, *J* = 11.5 Hz, 1H, Bn*H*), 4.83 (d, *J* = 12.0 Hz, 1H, Bn*H*), 4.76 (d, *J* = 11.4 Hz, 1H, Bn*H*), 4.70 (d, *J* = 12 Hz, 1H, Bn*H*), 4.33-4.22 (m, 2H, H-6), 4.13-

4.06 (m, 3H, H-3, H-4 , H-5), 4.00-3.96 (m, 1H, H-2), 3.78 (s, 3H. OCH_3), 2.60 (s, 1H, -OH), 1.96 (s, 3H, $COCH_3$) ^{13}C NMR (100 MHz, $CDCl_3$): δ 170.9 (C=O), 155.3, 150.9, 138.2, 138.1, 128.7, 128.6, 128.2, 128.1, 128.0, 118.7, 114.5, 97.2 (C-1), 77.4 (C-2), 75.6, 73.5, 73.2, 68.2 (C-3), 67.7 (C-4), 63.5 (C-6), 55.7 (C-5,), 20.9 ($COCH_3$) HRMS (ESI-TOF): calcd for $C_{29}H_{32}O_7Na^+$ $(M + Na)^+$, 531.1989; found 531.1982.

4,6-*O*-Benzylidene-3-*O*-chloroacetyl-2-(2,2,2-trichloroethoxycarbonyl-amino)-2-deoxy-β-D-galactopyranosyl-(1→4)-4-methoxyphenyl-6-*O*-acetyl-2,3-di-*O*-benzyl-α-D-galactopyranoside (4). A mixture of donor **6** (0.276 g, 0.44 mmol), acceptor **7** (0.150 g, 0.294 mmol), and molecular sieves (4 Å, 1 g) was stirred in anhydrous DCM (10 mL) for 1 h under a nitrogen atmosphere. The reaction mixture was cooled to 0 °C. Then, NIS (0.120 g, 0.529 mmol) and TMSOTf (7.9 μL, 0.044 mmol) were added to the reaction mixture and the solution was stirred at same temperature for 20 min after which TLC (40% EA-PE) showed complete consumption of the starting material. Then, the reaction was quenched by the addition of triethylamine and the solution was filtered through a pad of Celite-545 and washed successively with saturated $NaHCO_3$ and $Na_2S_2O_3$ solutions. The organic layer was dried over anhydrous Na_2SO_4 and the solvent was evaporated *in vacuo*. The crude product was purified by column chromatography using 30% EA/PE as eluent to afford the disaccharide **4** (0.252 g, 85%) as light yellow viscous liquid. $[\alpha]_D^{25}$= +70.74 (*c* = 0.36, $CHCl_3$); IR (cm^{-1}, $CHCl_3$) 2925, 1741, 1713, 1507, 1455, 1369, 1245, 1171, 1090, 1038, 823, 790, 745 NMR (400 MHz, $CDCl_3$): δ 7.48-7.28 (m, 15H, Ar*H*), 6.99 (d, *J* = 9.0 Hz, 2H, Ar*H*), 6.81 (d, *J* = 9 Hz, 2H, Ar*H*), 5.75 (d, *J* = 6.8 Hz, 1H, N*H*), 5.47 (s, 1H. C*H*Ph), 5.35 (d, *J* = 2.4 Hz, 1H, H-1), 5.05 (d, *J* = 11.0 Hz, 1H, Bn*H*), 4.87 (d, *J* = 11.5 Hz, 1H, CH'_2CCl_3), 4.83 (d, *J* = 12.2 Hz, 1H, Bn*H*), 4.81 (d, *J* = 12.2 Hz, 1H H-3'), 4.69 (d, *J* = 11.5 Hz, 1H, CH'_2CCl_3), 4.68 (d, *J* = 11.5 Hz, 1H, Bn*H*), 4.65 (d, *J* = 7.8 Hz, 1H, H-1'), 4.45 (d, *J* = 11.7 Hz, 1H, H-6), 4.39 (d, *J* = 12.2 Hz, 1H, Bn*H*), 4.25-4.01 (m,

11H, H-6,, H-4', H-2′, H-2, H-3, H-4, H-5, 2H-6', 2xOCOC*H*$_2$Cl), 3.77 (s, 3H, OCH_3), 3.36 (bs, 1H, H-5′), 1.88 (s, 3H, COC*H*$_3$), ^{13}C NMR (100 MHz, $CDCl_3$): δ 170.0 (C=O), 167.2 (C=O), 155.3, 154.4, 150.8, 138.2, 137.9, 137.8, 137.5, 129.4, 129.1, 128.8, 128.7, 128.4, 128.4, 128.1, 127.9, 126.5, 118.8, 114.6, 101.5 (C-1'), 101.1 (Ph*C*), 97.2 (C-1) , 95.8 (CCl_3), 78.2 (C-3′), 77.4 (C-4′), (C-2), 76.6 (C-3), 74.9 (*C*H_2Ph), 74.3 (*C*H_2Ph), 74.3 (C-3'), 74.0 (C-6′), 72.8 (C-4), 68.9 (*CH′*$_2$$CCl_3$), 68.7 (C-2), (C-5), 66.5 (C-5′), 64.2 (C-6), 55.8 (-O*C*H_3), 52.4 (C-2′), 41.0 (O*C*H_2Cl), 20.9 (CH_3). HRMS (ESI-TOF): calcd for $C_{47}H_{49}Cl_4NO_{15}Na^+$ $(M + Na)^+$, 1030.1749; found, 1030.1746.

4,6-*O*-Benzylidene-2-(2,2,2-trichloroethoxycarbonylamino)-2-deoxy-β-D-galactopyranosyl-(1→4)-4-methoxyphenyl-6-*O*-acetyl-2,3-di-*O*-benzyl-α-D-galactopyranoside (5). To a stirred solution of **4** (0.200 g, 0.2 mmol) in DCM/MeOH 2:3 (2 mL), thiourea (0.076 g, 1 mmol) and 2,4,6-collidine (0.031 mL, 0.23 mmol) were added sequentially. The reaction mixture was refluxed for 12 h when TLC (45% EA/PE) showed complete conversion of the starting material. The reaction mixture was diluted with DCM and the organic layer was washed with water. The organic layer was separated and the aqueous part was extracted thrice with saturated aqueous $NaHCO_3$. Then, the combined organic layer was washed with brine, dried over anhydrous Na_2SO_4, filtered, and concentrated *in vacuo*. The crude product was purified by column chromatography using 40% ethyl EA/PE as eluent to afford **5** (0.160 g, 87%) as a yellowish liquid. $[\alpha]_D^{25}$ = 50.36 (*c* = 0.13), $CHCl_3$ IR (cm^{-1}, $CHCl_3$) 3425, 2919, 1736, 1730, 1713, 1571, 1507, 1453, 1369, 1244, 1089, 1037, 824, 739, 698 ^{1}H NMR (400 MHz, $CDCl_3$): δ 7.50-7.48 (m, 2H, Ar*H*), 7.44-7.28 (m, 13H, Ar*H*), 6.97 (d, *J* = 9.0 Hz, Ar*H*), 6.80 (d, *J* = 9.1 Hz, 2H, Ar*H*), 6.44 (d, *J* = 5.0 Hz, 1H, N*H*), 5.53 (s, 1H, C*H*Ph), 5.35 (d, *J* = 3.3 Hz, 1H, H-1), 5.11 (d, *J* = 11.0 Hz, 1H, Bn*H*), 4.91 (d, *J* = 11.5 Hz, 1H, Bn*H*), 4.78 (d, *J* = 12.0 Hz, 1H, H-1′), 4.71 (d, *J* = 4.68 Hz, 1H, H-6′), 4.69 (d, *J* = 5.1 Hz, 1H H-6′), 4.63 (d, *J* = 12.0 Hz, 1H Bn*H*), 4.49 (dd, *J* = 11.6 and 2.1 Hz, 1H, H-6),

4.44 (d, J = 8.4 Hz, 1H, H-1′), 4.27-4.12 (m, 4H, H-3, H-4, H-6, H-5), 4.08-4.02 (m, 5H, H-2, H-3′, H-4′, CH'_2CCl_3), 3.96-3.90 (m, 1H, H-2′,), 3.77 (s, 3H, -OCH_3), 3.45-3.40 (m, 1H, -O*H*), 3.31 (bs, 1H, H-5′), 1.88 (s, 3H, COCH_3) ^{13}C NMR (100 MHz, $CDCl_3$): δ 171.1, 156.9, 155.3, 150.8, 138.1, 137.7, 137.6, 139.3, 129.0, 128.7, 128.5, 128.4, 128.1, 127.9, 126.6, 118.8, 114.6, 102.2 (C-1′), 101.4, 97.1 (C-1), 95.5, 78.1, 77.6 (C-3), (C-3′), 77.6 (C-4′), 75.2 (CH_2Ph), 75.0 (CH_2Ph), 74.8 (C-5) , 74.4 (C-1′), 74.0 (C-6′), 69.0 (CH'_2CCl_3) , 68.5 (C-2), (C-4), 66.9 (C-5′), 64.2 (C-6), 55.8 (COCH_3), 55.3 (C-2′), 20.9 HRMS (ESI-TOF): calcd for $C_{45}H_{48}Cl_3NO_{14}Na^+$ (M + Na)$^+$, 954.2033; found, 954.2016.

2-*O*-Acetyl-3-*O*-benzyl-4,6-*O*-benzylidene-β-D-galactopyranosyl-(1→3)-4,6-*O*-benzylidene-2-(2,2,2-trichloroethoxycarbonylamino)-2-deoxy-β-D-galactopyranosyl-(1→4)-4-methoxyphenyl-6-O-acetyl-2,3-di-*O*-benzyl-α-D-galactopyranoside (2). A mixture of donor **3** (0.175 g, 0.345 mmol), acceptor **5** (0.140 g, 0.15 mmol), and molecular sieves (4 Å, 1 g) was stirred in anhydrous DCM (10 mL) for 1 hour under nitrogen atmosphere. Then, the mixture was cooled to 0 °C and NIS (0.060 g, 0.26 mmol) and TMSOTf (4.0 μL, 10.0 mmol) were added. The solution was stirred at same temperature for 15 min, after which TLC (50% EA/PE) showed complete consumption of the donor. The reaction was quenched by addition of triethylamine. The solution was filtered through a pad of Celite-545 and washed successively with saturated aqueous $NaHCO_3$ and $Na_2S_2O_3$ solutions. The organic layer was dried over anhydrous Na_2SO_4 and concentrated *in vacuo*. The crude product was purified by column chromatography using 40% EA/PE as eluent to afford the trisaccharide **2** (0.175 g, 89%) as colorless sticky liquid. $[\alpha]_D^{25}$ = +52.7 (c = 0.21, $CHCl_3$); IR (cm^{-1}, $CHCl_3$) 2924, 2854, 1741, 1738, 1506, 1455, 1403, 1368, 1236, 1088, 1055, 821, 792, 736, 697. ^{1}H NMR (400 MHz, $CDCl_3$): δ 7.52-7.47 (m, 4H, Ar*H*), 7.42-7.27 (m, 21H, Ar*H*), 6.99 (d, 9.0 Hz, 2H, Ar*H*), 6.80 (d, J = 9.0 Hz, 2H, Ar*H*), 5.53 (s,

1H, C*H*Ph), 5.49 (s, 1H, C*H*Ph), 5.45 (d, *J* = 7.3 Hz, 1H, N*H*), 5.36 (d, *J* = 2.2 Hz, 1H, H-1), 5.31 (dd, *J* = 10.0, 8.1 Hz, 1H H-2″), 4.85 (d, *J* = 8.5 Hz, 1H, H-1''), 4.84 (d, *J* = 11.7 Hz, 1H, C*H*'$_2$CCl$_3$), 4.77 (d, *J* = 12 Hz, 1H, Bn*H*), 4.71-4.65 (m, 3 H, C*H*'$_2$CCl$_3$, Bn*H*, Bn*H),* 4.61 (d, *J* = 7.8 Hz, 1H, H1'), 4.58 (d, *J* = 12.5 Hz, 1H, Bn*H*), 4.45 (dd, *J* = 12.1 and 2.8 Hz, 1H, H-6), 4.40 (d, *J* = 12.2 Hz, 1H, H-6'), 4.34-4.27 (m, 4 H, Bn*H*, H-4, H-6', H-6''), 4.22-3.94, (m, 9H, Bn*H*, H-6, H-6'', H-2, H-2′, H-3′, H-4', H-4″, H-5), 3.76 (s, 3H, -OCH$_3$), 3.73-3.66 (m, 1H, H-3), 3.42 (dd, *J* = 10.0 and 3.3 Hz, 1H, H-3″), 3.36 (bs, 1H, H-5''), 3.31 (bs, 1H, H-5′), 2.02 (s, 3H, C*H*$_3$), 1.84 (s, 3H, C*H*$_3$). ^{13}C NMR (100 MHz, CDCl$_3$): δ 171.0 (*C*=O), 169.7 (*C*=O), 155.2, 154.1, 150.8, 138.6, 138.5, 138.1, 137.9, 137.6, 129.2, 129.0, 12 9.0, 128.6, 128.6, 128.6, 128.4, 128.3, 128.2, 128.0, 128.0, 128.0, 127.8, 127.7, 126.6, 126.5, 118.9, 114.5, 101.2 (*C*HPh), 101.0 (*C*HPh),100.9 (*C*-1''), 100.5 (*C*-1'), 97.2 (C-1), 95.8 (CCl$_3$)78.2 (C-2), 77.3 (C-5’)76.2 (C-5″), 75.3 (C-2′), 74.8 (C-4″), 74.0 (*C*H$_2$Ph), 73.7 (*C*H$_2$Ph), 73.6 (*C*H$_2$Ph), 73.5 (*C*-1′), 72.9 (C-4′), 71.3 (C-6″), 69.9 (C-2″), 69.3 (C-6′), 69.1 (C-3″), 69.0 (*C*-1″), 66.7 (C-4) 66.5 (C-5), 64.5 (C-6), 55.7 (CO*C*H$_3$), 53.9 (C-3), 21.1 (CH$_3$), 20.9 (CH$_3$). HRMS (ESI-TOF): calcd for $C_{67}H_{70}Cl_3NO_{20}Na^+$ (M + Na)$^+$, 1336.3449; found, 1336.3473.

2-*O*-Acetyl-3-*O*-benzyl-4,6-*O*-benzylidene-D-galactopyranose (11). To a solution of compound **3** (0.150 g, 0.3 mmol) in acetone/water 4:1 (7.5 mL), TCCA (0.068 g, 0.3 mmol) was added at 0 °C. The mixture was stirred for 20 min at room temperature, after which TLC (40% EA/PE) showed complete consumption of the starting material. Then, the reaction mixture was diluted with DCM and washed with saturated NaHCO$_3$ solution and water. The organic layer was dried over anhydrous Na$_2$SO$_4$ and concentrated *in vacuo*. The crude product was then purified by column chromatography using 60% EA/PE to yield a sticky liquid of **11** (0.116 g, 98%) as a mixture of of α/β-isomers (1:1.2). IR (cm^{-1}, CHCl$_3$) 3417, 2923, 2853, 1708, 1373, 1267, 776, 666. ^{1}H NMR (400 MHz, CDCl$_3$): δ 7.55-7.53 (m, 3H, Ar*H*), 7.38-7.29 (m, 13H,

Ar*H*), 5.55 (d, *J* = 3.2 Hz, 1H), 5.50 (s, 1H), 5.46 (s, 1H), 5.30 (dd, *J* =10.4 and 3.4 Hz, 1H), 5.20 (dd, *J* = 9.8 and 8.3 Hz, 1H) 4.73-4.64 (m, 3H), 4.56 (d, *J* = 8.0 Hz, 1H), 4,34-4.16 (m, 4H), 4.04-3.97 (m, 3H), 3.82 (s, 1H), 3.61 (dd, *J* = 10.0 and 3.4 Hz, 1H), 3.50-3.40 (m, 2H), 2.14-2.09 (m, 5H), 1.84 (s, 1H, -OH) ^{13}C NMR (100 MHz, $CDCl_3$): δ 171.7, 170.6, 169.8, 138.6, 138.5, 137.9, 137.8, 137.6, 137.6, 129.1, 129.0, 128.6, 128.6, 128.5, 128.3, 128.3, 128.2, 128.0, 128.0, 127.8, 127.7, 127.6, 126.4, 126.3, 126.3, 101.0, 101.0, 96.2, 92.5, 91.2, 76.4, 74.3, 73.3, 73.2, 73.0, 73.0, 71.9, 71.6, 71.5, 70.4 (C-5), 69.4 (C-6), 69.2, 66.9, 66.6, 64.9, 62.5, 21.2, 21.2. HRMS (ESI-TOF): calcd for $C_{22}H_{24}O_7Na^+$ (M + Na)$^+$, 423.1414; found, 423.1404.

2-*O*-Acetyl-3-*O*-benzyl-4,6-*O*-benzylidene-D-glucopyranosyl trichloro-acetimidate (12). To a solution of compound **11** (0.116 g, 0.28 mmol) and CCl_3CN (0.0435 mL, 0.43 mmol) in anhydrous DCM (2 mL), DBU (0.012 mL, 0.09 mmol) was added at −5 °C. The reaction mixture was stirred at the same temperature for 5 h after which TLC (70% EA/PE) showed complete consumption of the starting material. Then, the solvent was removed *in vacuo* and the resulting crude product was purified by flash column chromatography using 20% EA/PE as eluent to furnish **12** as a colorless sticky syrup (0.147 g, 93%). IR (cm^{-1}, $CHCl_3$) 1632, 1071, 1038, 668. ^{1}H NMR (400 MHz, $CDCl_3$): δ 8.58 (s, 1H, N*H*), 7.55-7.53 (m, 2H, Ar*H*), 7.40-7.28 (m, 8H, Ar*H*), 6.66 (d, *J* = 3.3 Hz, 1H, H-1), 5.53 (s, 1H, Ph*H*), 5.52 (s, 1H, Ph*H*), 5.50 (d, *J* = 3.4 Hz, 1H), 4.77-4.70 (m, 2H, H-6), 4.34 (d, *J* = 2.9 Hz, 1H, H-4), 4.30 (dd, *J* = 12.6 and 1.4 Hz, 1H, Bn*H*), 4.11 (dd, *J* = 10.5 and 3.3 Hz, 1H, H-3), 4.03 (dd, *J* = 12.6 and 1.6 Hz, 1H, Bn*H*), 3.87 (bs, 1H, H-5), 2.00 (s, 3H, CH_3) ^{13}C NMR (100 MHz, $CDCl_3$): δ 170.3 (C=O), 160.9, 137.9, 137.4, 129.3, 128.5, 128.4, 128.1, 126.5, 126.3, 101.3, 94.9 (C-1), 91.2, 73.9, 72.8, 72.0, 69.1, 69.0, 65.4, 29.8, 20.8 (COCH_3) HRMS (ESI-TOF): calcd for $C_{24}H_{24}Cl_3NO_3K^+$ (M + K)$^+$, 582.025; found, 582.7082.

One-pot synthesis of compound 2. A mixture of donor **12** (0.147 g, 0.270 mmol), acceptor **9** (0.128 g, 0.233 mmol), and molecular sieves (4 Å, 0.500 g) was stirred in anhydrous DCM (5 mL) for 30 min at room temperature. Then, the mixture was cooled to −20 °C. After 10 min, TMSOTf (5 µL, 0.027 mmol) was added and the reaction mixture was further stirred at −20 °C for 2 h when complete consumption of both starting materials was observed (TLC, 60% EA-PE). An aliquot was withdrawn from the reaction mixture and analysis by HRMS revealed the formation of the disaccharide [HRMS (ESI-TOF): calcd for $C_{24}H_{24}Cl_3NO_3Na^+$ $(M + Na)^+$, 952.1699; found, 952.1704. The above reaction mixture was warmed to room temperature and stirred for 30 min. Then, the acceptor **7** (0.123 g, 0.21 mmol) was added as a solution in DCM (1 mL), to the same reaction vessel and the mixture was stirred for 30 min at room temperature. Thereafter, the reaction mixture was cooled to 0 °C and NIS (0.072g, 0.32 mmol) and TMSOTf (5 µL, 0.027 mmol) was added. The solution was stirred at 0 °C for a further 15 min, after which TLC (60% EA/PE) showed complete consumption of the starting materials. Then, the reaction was quenched by addition of triethylamine and the mixture was filtered through a celite bed. The filtrate was washed successively with saturated aqueous $NaHCO_3$ and $Na_2S_2O_3$ solutions, dried over anhydrous Na_2SO_4, and concentrated *in vacuo*. The crude product was purified by column chromatography using 40% EA/PE as eluent to afford the trisaccharide **2** (0.223 g, 73%) as colorless liquid. The structure of **2** was confirmed by comparison of its spectral data with those of the previously synthesized compound **2**.

β-D-Galactopyranosyl-(1→3)-2-deoxy-2-acetylamino-β-D-galactopyranosyl-(1→4)-4-methoxyphenyl-α-D-galactopyranoside (1). To a solution of **2** (0.078 g, 0.06 mmol) in dry THF (4 mL) at 0 °C, activated zinc dust (0.234 g, 3.6 mmol), acetic acid (0.24 mL, 4.1 mmol), and acetic anhydride (0.16 mL, 1.7 mmol) were added. The reaction mixture was warmed to room temperature and stirred

for 3 h after which TLC (60% EA/PE) showed complete consumption of the starting material. Then, the reaction mixture was filtered through a pad of Celite-545. The filtrate was quenched with saturated $NaHCO_3$. The THF layer was collected and the aqueous layer was extracted thrice with EA. The combined organic layer was dried over anhydrous Na_2SO_4 and concentrated *in vacuo*. Then any residual solvent was removed by co-evaporation with toluene after which the crude product was dried under vacuum.

The crude product was dissolved in MeOH (2 mL) and NaOMe (4 mg, 0.074 mmol) was added. Then, the reaction mixture was stirred at room temperature for 2 h after which TLC (2% MeOH/EA) showed complete consumption of the starting material. Then, the reaction mixture was neutralized by Amberlite-120 H^+ resin, filtered, and the filtrate was concentrated *in vacuo*. The viscous liquid was co-evaporated with toluene to remove any residual solvent and further dried under vacuum.

The crude product obtained above was redissolved in methanol (5 mL) and $Pd(OH)_2/C$ (0.156 g, 20% Pd content) was added. The reaction mixture was stirred under an atmosphere of hydrogen for 24 h. Then, the reaction mixture was filtered through a Celite-545 pad. The filtrate was concentrated *in vacuo* to afford **1** as white foam (30 mg, 68%). $[\alpha]_D^{25}$ = +32.2 (*c* = 0.23, MeOH); IR (cm^{-1}, CH_3OH) 3535, 2923, 1636, 1507, 1383, 1214, 1035. ^1H NMR (400 MHz, $CDCl_3$): δ 7.08 (d, *J* = 8.8 Hz, 1H, Ar*H*), 6.94 (d, *J* = 8.8 Hz, 1H, Ar*H*), 5.46 (d, *J* = 3.1 Hz, 1H, H-1), 4.66 (d, *J* = 8.5 Hz, 1H (H-1′), 4.41 (d, *J* = 7.6 Hz, 1H, H-1″) , 4.17-4.06 (4H, m H-4, H-4′, H-4″, H-6), 4.00 (m, 1H, H-6), 3.92-3.57 (16H, m, H-2, H-2′, H-2″, H-3, H-3′, H-3″, , H-5, H-5′, H-5″ , H-6′, H-6″, OCH_3), 3.49 (t, *J* = 8.7 Hz, 1H, H-2″), 2.02 (s, 3H, CH_3) ^{13}C NMR (100 MHz, $CDCl_3$): δ174.9, 154.5, 150.3, 118.9, 114.9, 104.7 (C-1″), 102.5 (C-1′) , 98.4 (C-1), 79.6, 76.8, 74.9, 74.4, 72.3, 70.8, 69.1, 68.4, 68.2, 67.9, 63.5 (C-6′), 60.9 (C-6″), 60.4 (C-6), 55.6,

51.4, 22.3 (*C*H_3CO) HRMS (ESI-TOF): calcd for $C_{47}H_{49}Cl_4NO_{15}Na^+$ $(M + Na)^+$, 674.2267; found, 674.2368.

References

1) Armarego, W. L. F. *Purification of laboratory chemicals / W.L.F. Armarego and D.D. Perrin*, Butterworth Heinemann, Oxford; Boston, **1996.**
2) Podilapu, A. R.; Kulkarni, S. S.; *Org. Lett.* **2017**, *19*, 5466-5469.
3) Sun, B.; Yang, B.; Huang, X. *Sci. China Chem.* **2012**, *55*, 31-35.
4) Wu, X.; McFall-Boegeman, H.; Rashidijahanabad, Z.; Liu, K.; Pett, C.; Yu, J.; Schorlemer, M.; Ramadan, S.; Behren, S.; Westerlind, U.; Huang, X. *Org. Biomol. Chem.* **2021**, *19*, 2448-2455.
5) Emmadi, M.; Kulkarni, S. S. *Org. Biomol. Chem.* **2013**, *11*, 3098-3102.
