# Supplementary material for: Total synthesis of the O-antigen repeating unit of Providencia stuartii O49 serotype through linear and one-pot assemblies
Source: Beilstein J Org Chem. 2021 Dec 13;17:2915–21. doi: 10.3762/bjoc.17.199 (PMC8685571; doi:10.3762/bjoc.17.199)
Supplement: File 2 — Copies of 1H and 13C NMR spectra of all known and new compounds synthesized. [file Beilstein_J_Org_Chem-17-2915-s002.pdf]

BEILSTEIN JOURNAL OF ORGANIC CHEMISTRY

Supporting Information

for

Total synthesis of the O-antigen repeating unit of *Providencia stuartii* O49 serotype through linear and one-pot assemblies

Tanmoy Halder and Somnath Yadav

Beilstein J. Org. Chem. **2021,** *17,* 2915–2921. doi:10.3762/bjoc.17.199

Copies of ^{1}H and ^{13}C NMR spectra of all known and new compounds synthesized

License and Terms: This is a supporting information file under the terms of the Creative Commons Attribution License (https://creativecommons.org/licenses/by/4.0). Please note that the reuse, redistribution and reproduction in particular requires that the author(s) and source are credited and that individual graphics may be subject to special legal provisions.
The license is subject to the *Beilstein Journal of Organic Chemistry* terms and conditions: (https://www.beilstein-journals.org/bjoc/terms)

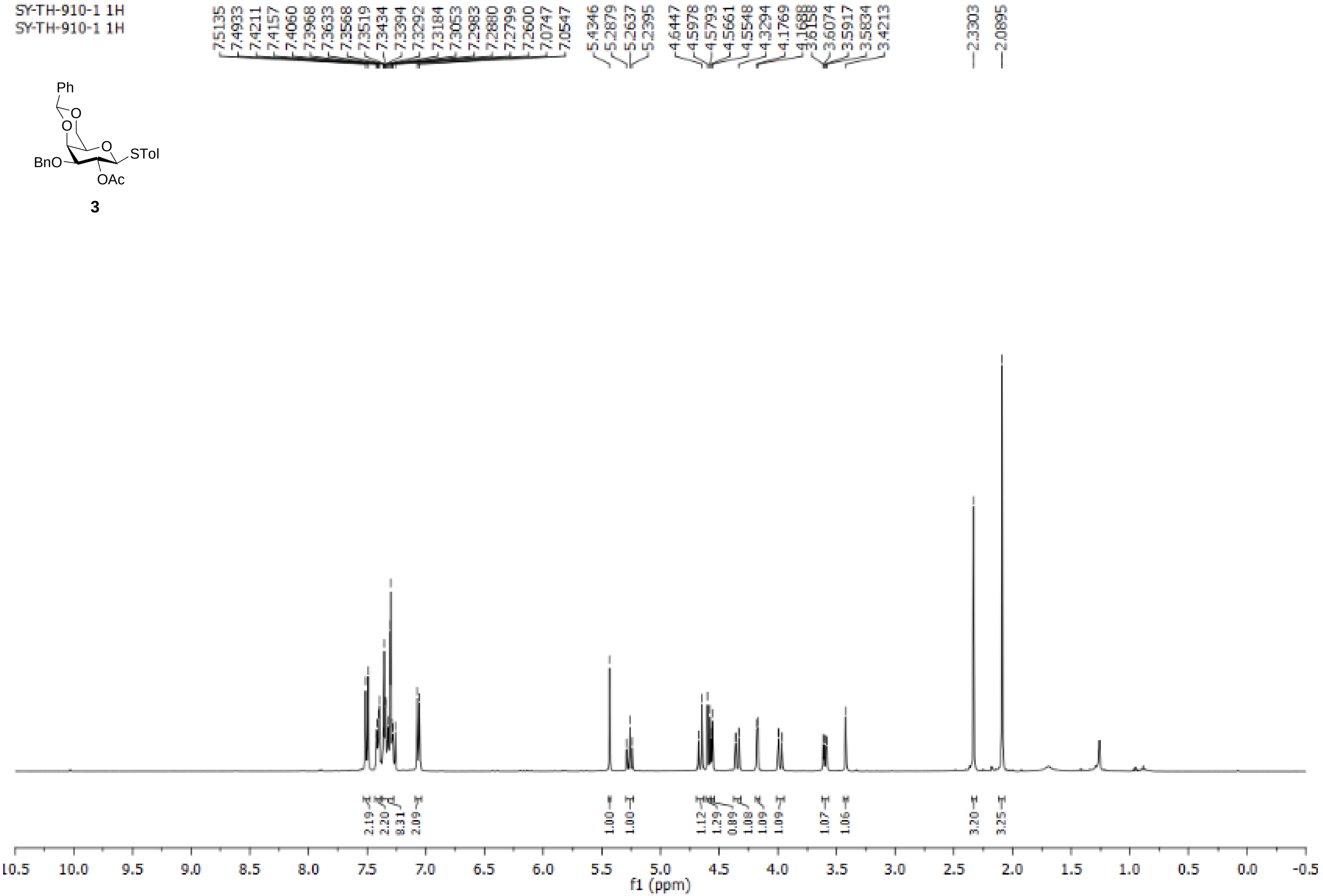

Figure S1: ^{1}H NMR spectrum (400 MHz, $CDCl_3$) of compound **3**.

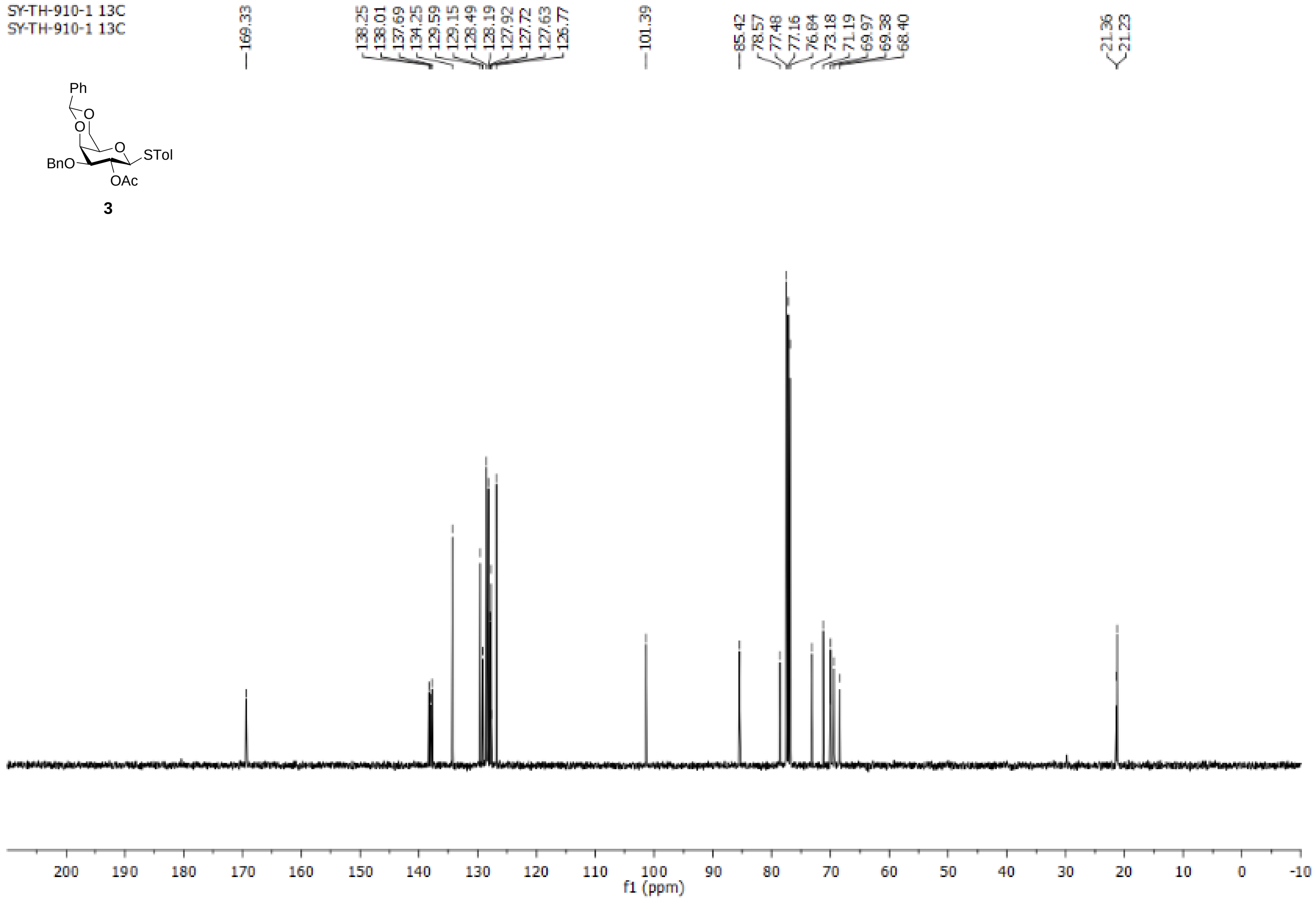

Figure S2: ^{13}C NMR spectrum (100 MHz, $CDCl_3$) of compound **3**.

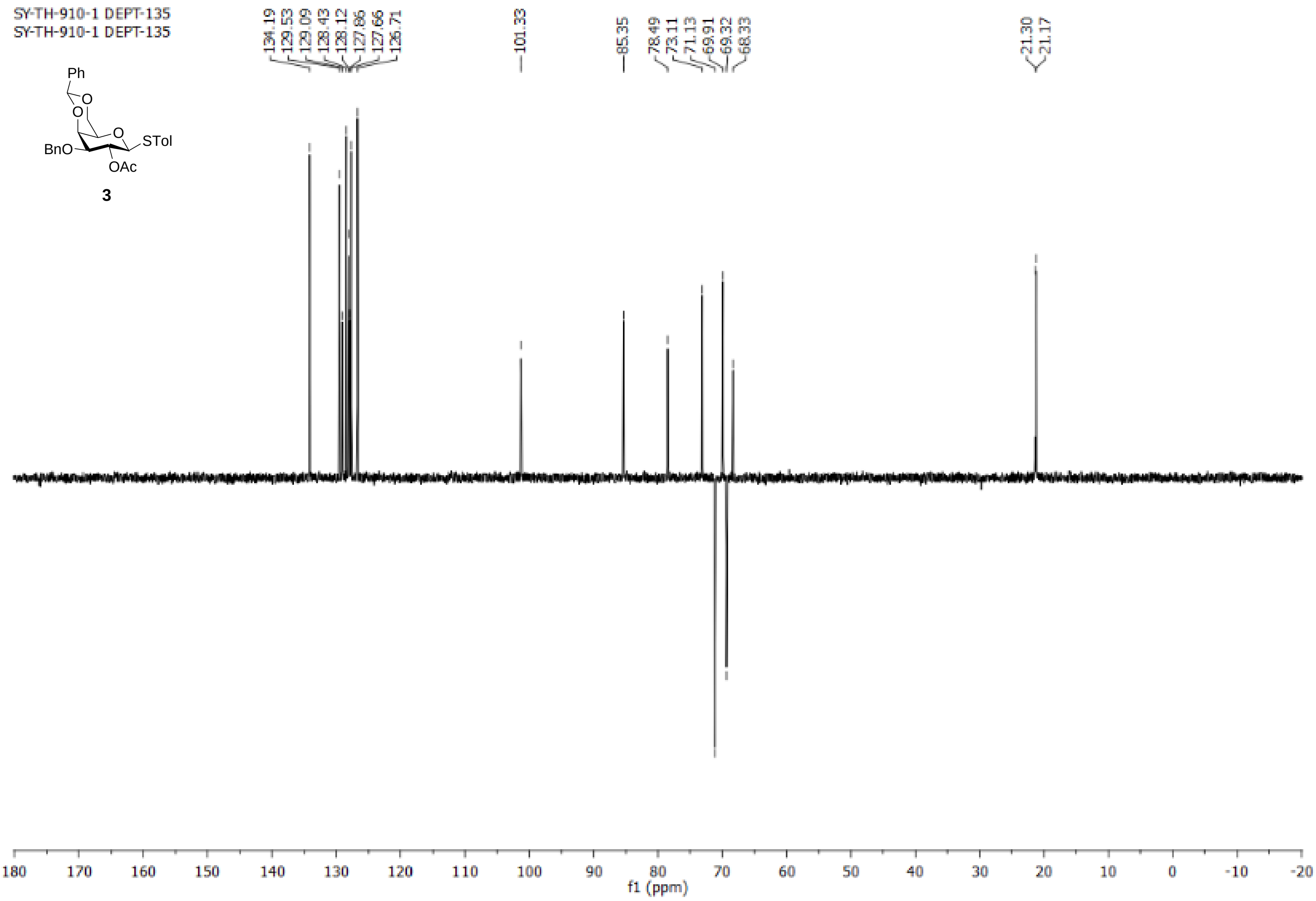

Figure S3: DEPT-135-NMR spectrum (100 MHz, $CDCl_3$) of compound **3**.

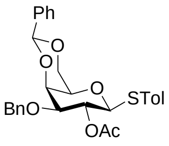

3

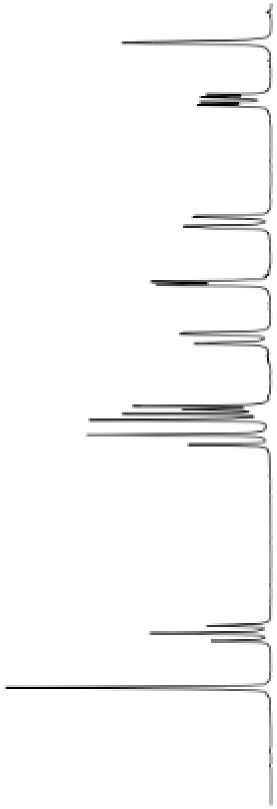

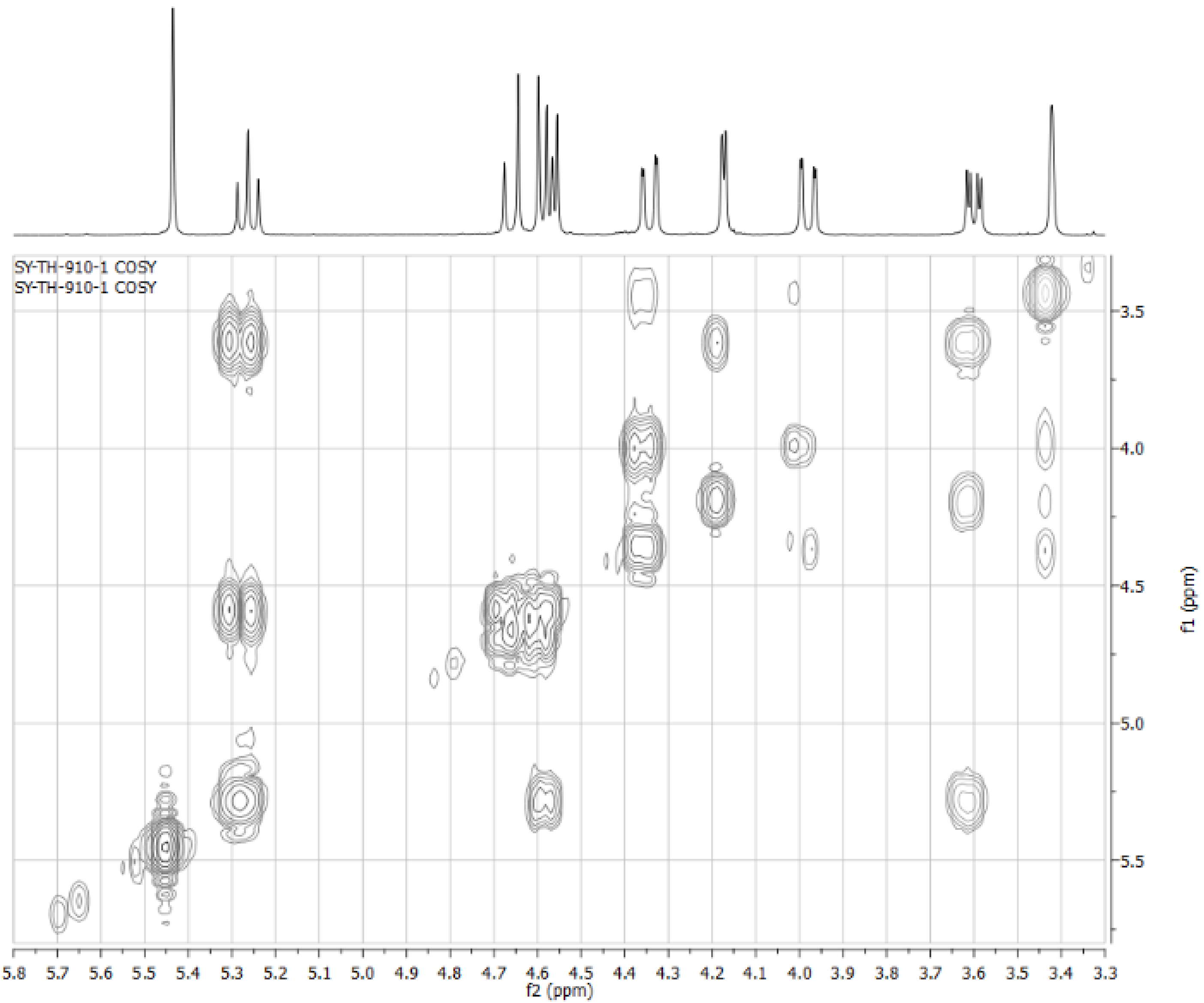

Figure S4: ^{1}H,^{1}H-COSY-NMR spectrum (400 MHz, $CDCl_3$) of compound **3**.

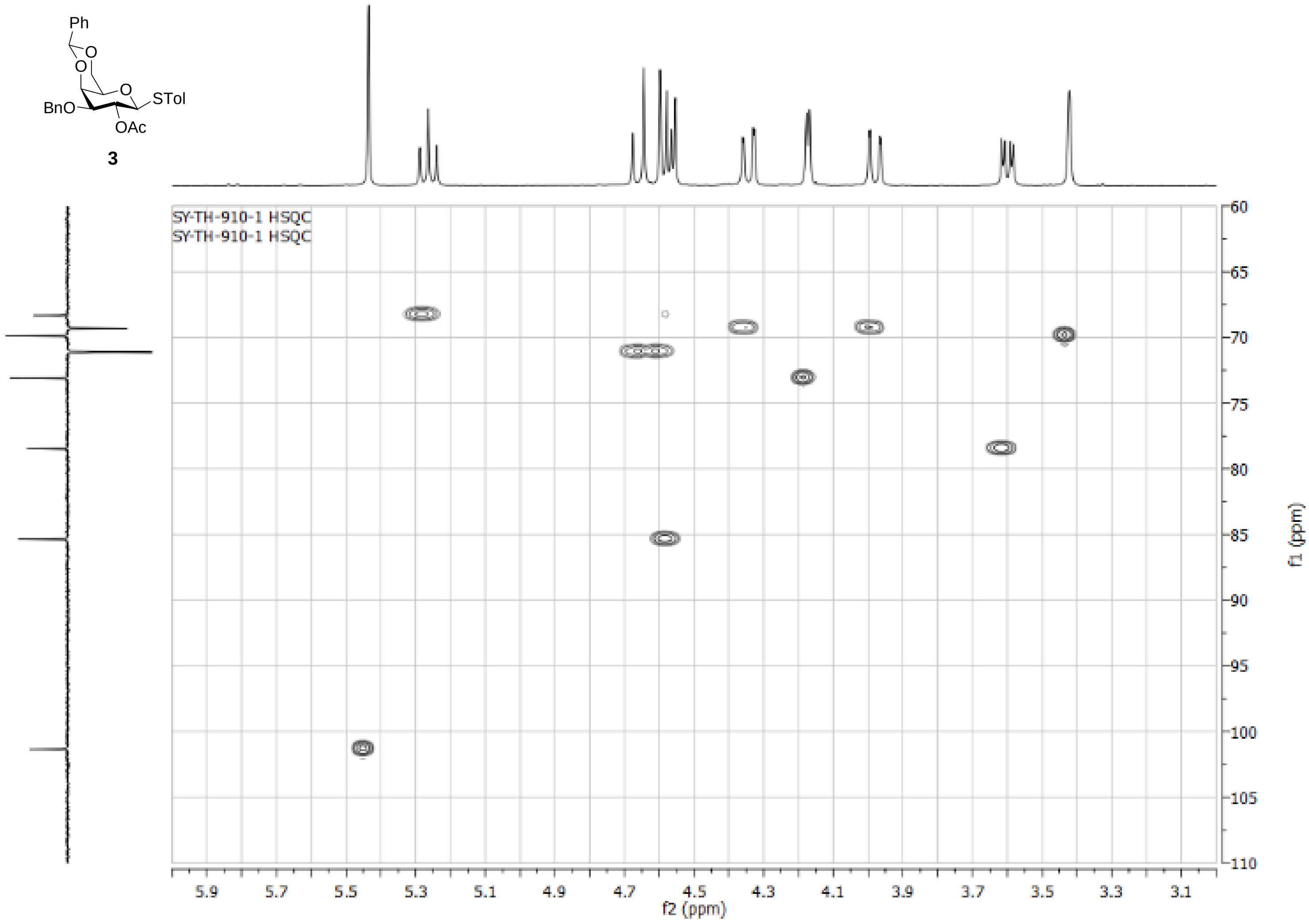

Figure S5: HSQC-NMR spectrum (400 MHz, $CDCl_3$) of compound **3**.

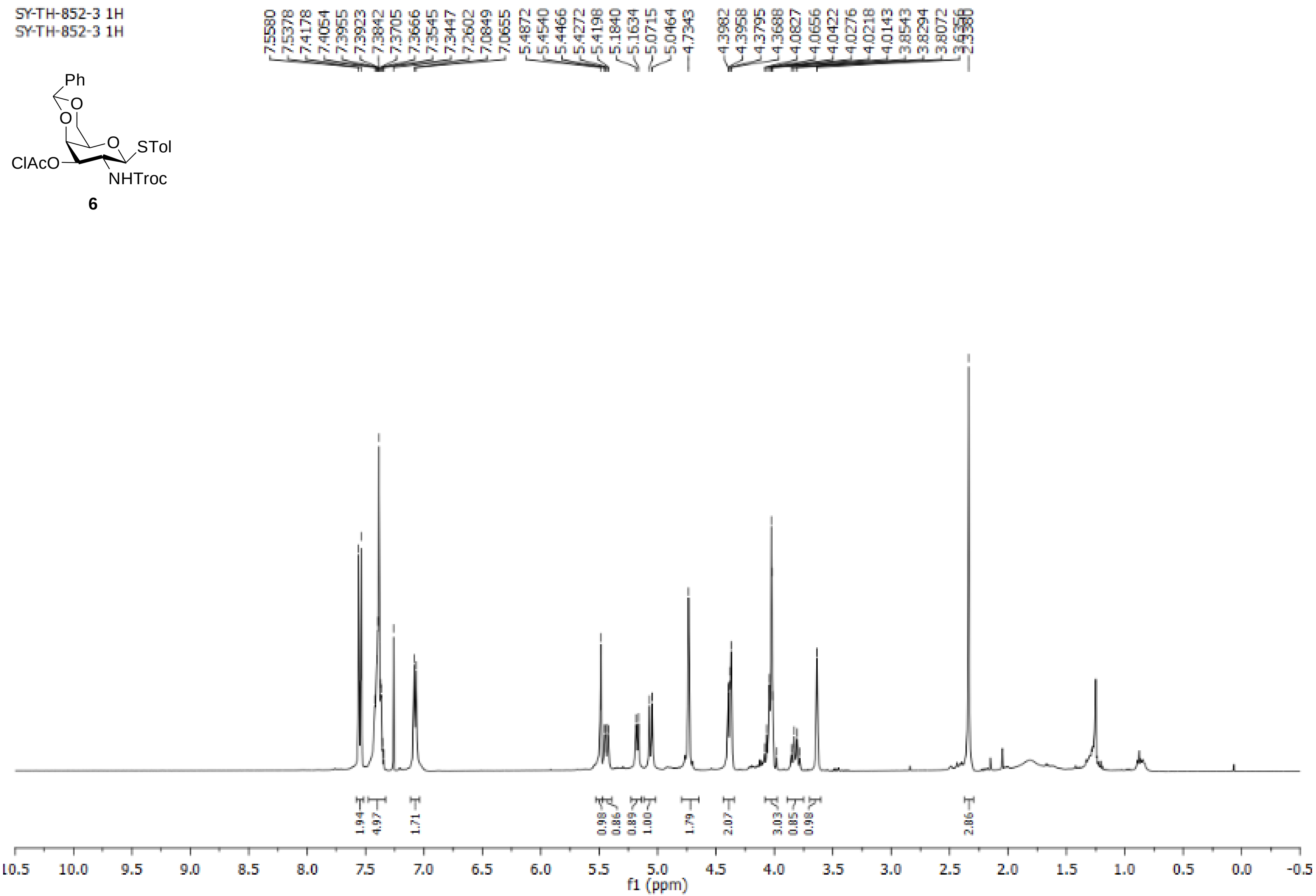

Figure S6: ^{1}H NMR spectrum (400 MHz, $CDCl_3$) of compound **6**.

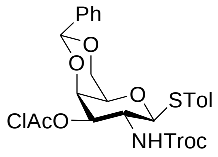

6

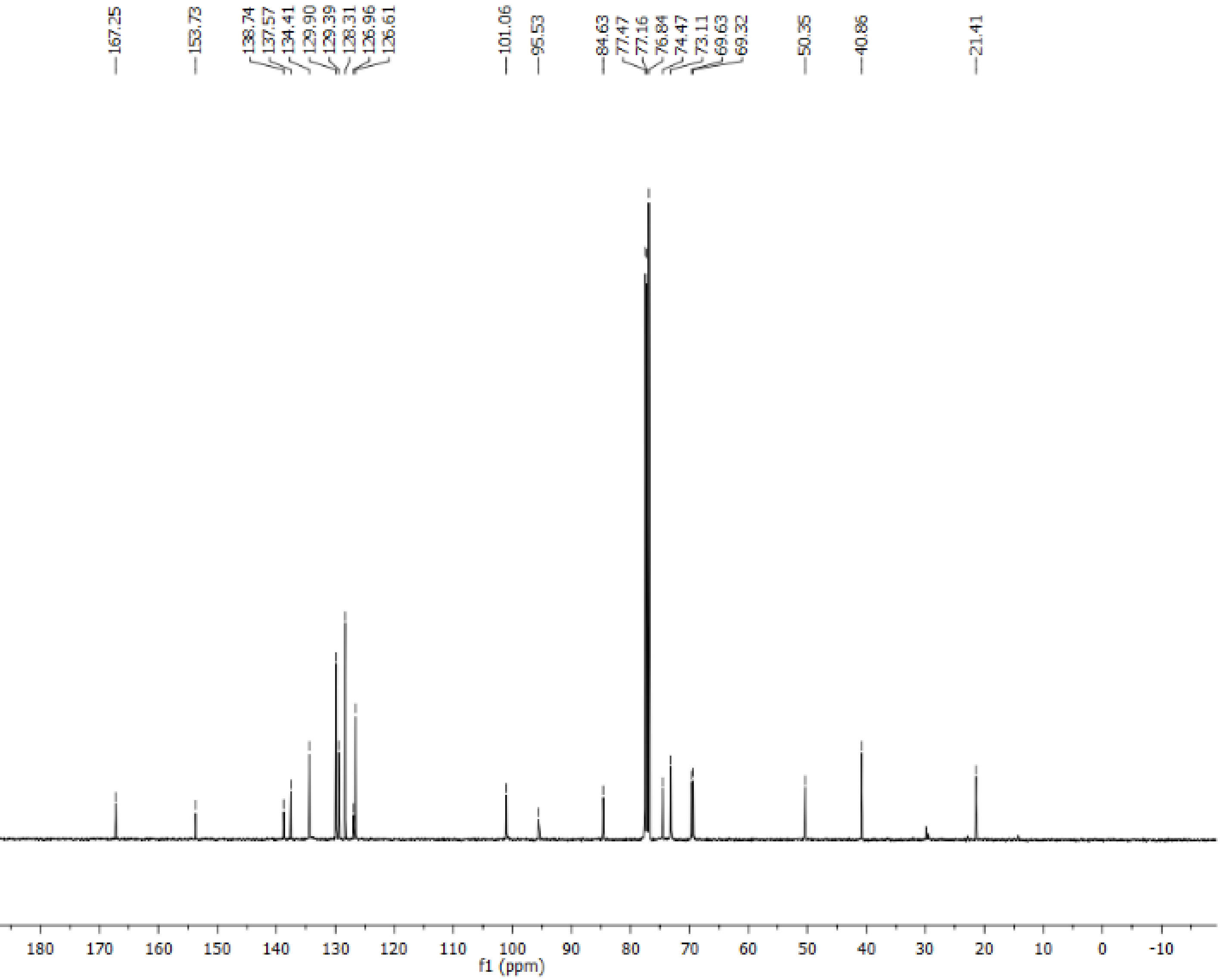

Figure S7: ^{13}C NMR spectrum (100 MHz, $CDCl_3$) of compound **6**.

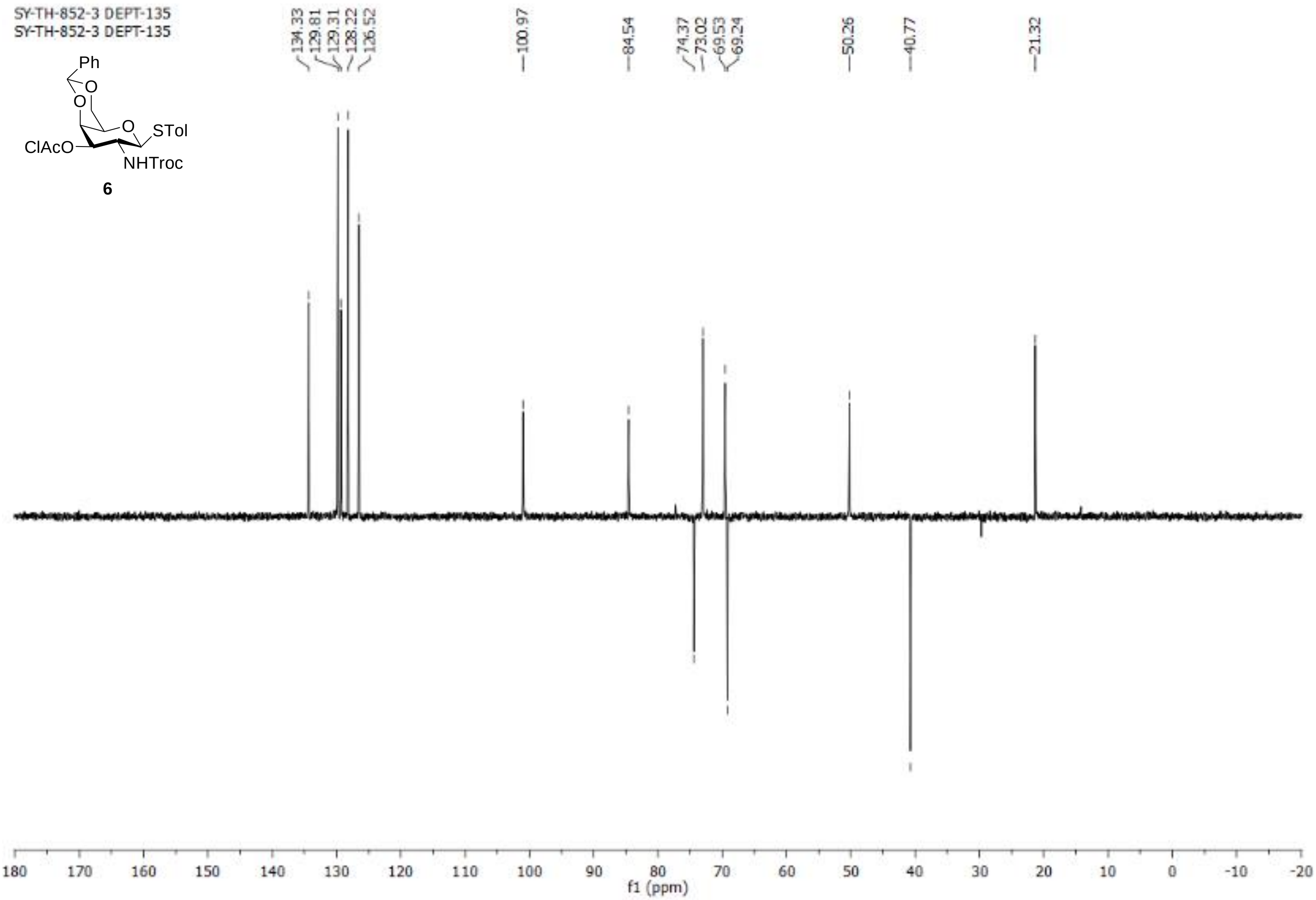

Figure S8: DEPT-135-NMR spectrum (100 MHz, $CDCl_3$) of compound **6**.

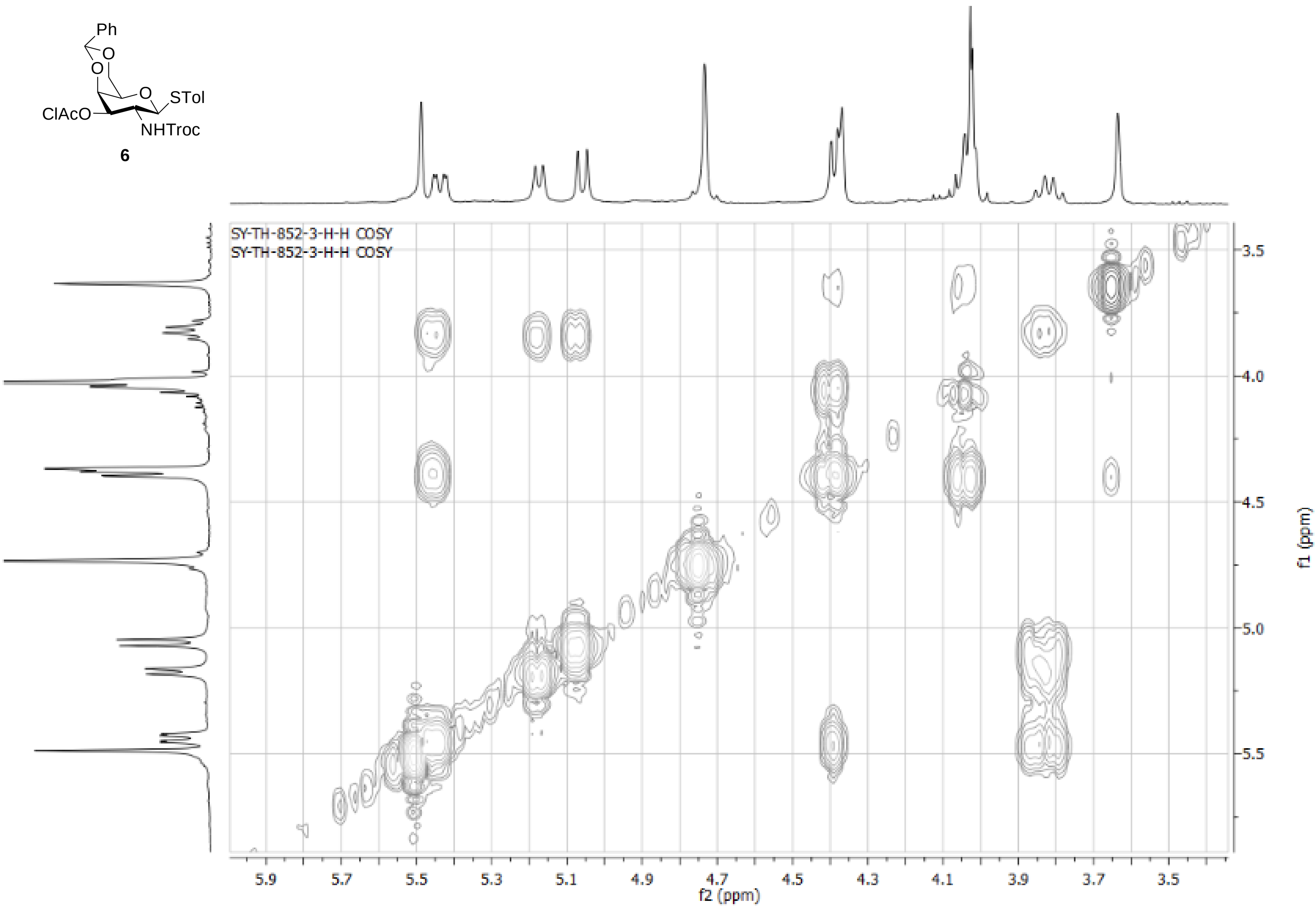

Figure S9: $^{1}H,^{1}H$-COSY-NMR spectrum (400 MHz, $CDCl_3$) of compound **6**.

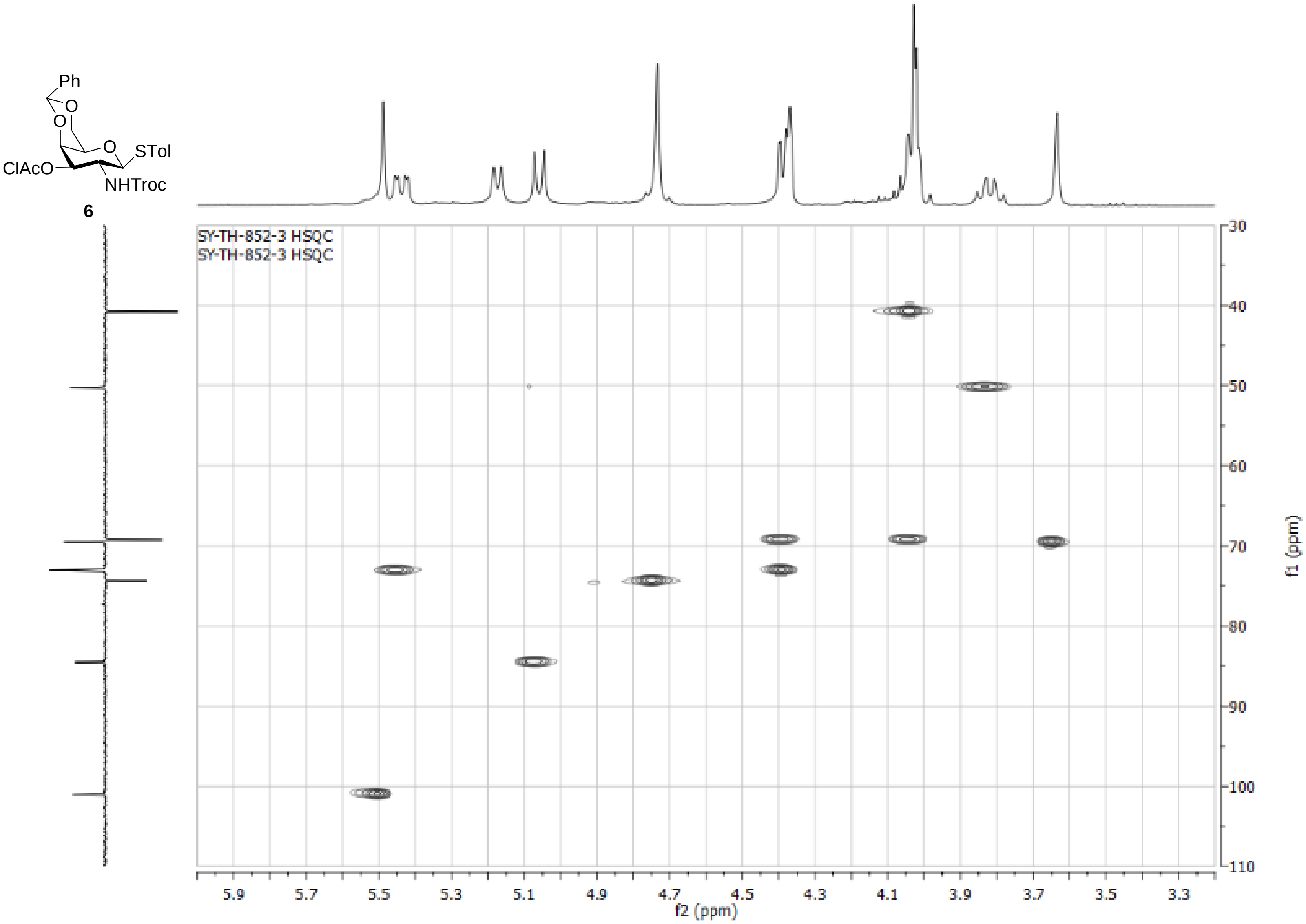

Figure S10: HSQC-NMR spectrum (400 MHz, $CDCl_3$) of compound **6**.

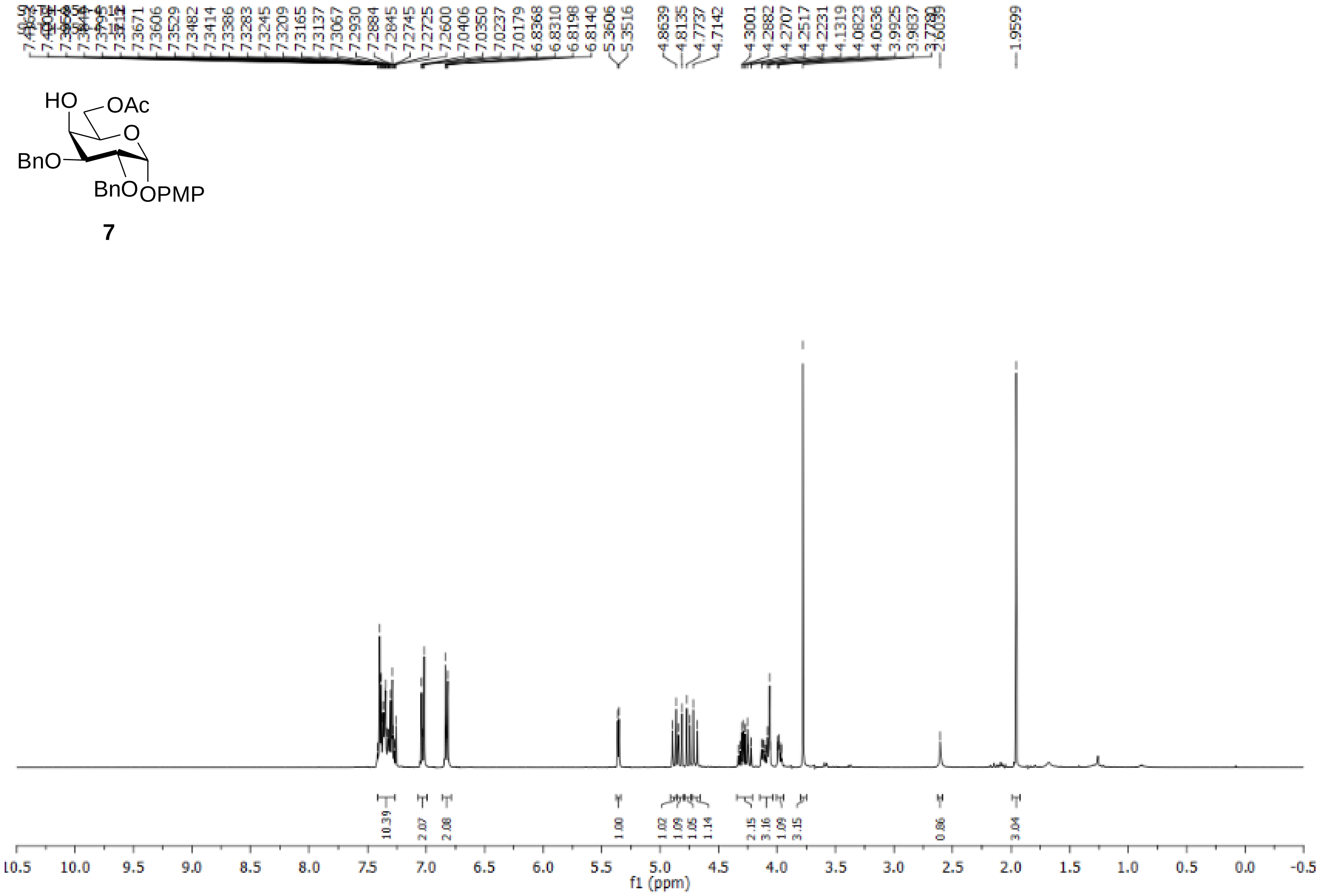

Figure S11. ^{1}H NMR spectrum (400 MHz, $CDCl_3$) of compound **7**.

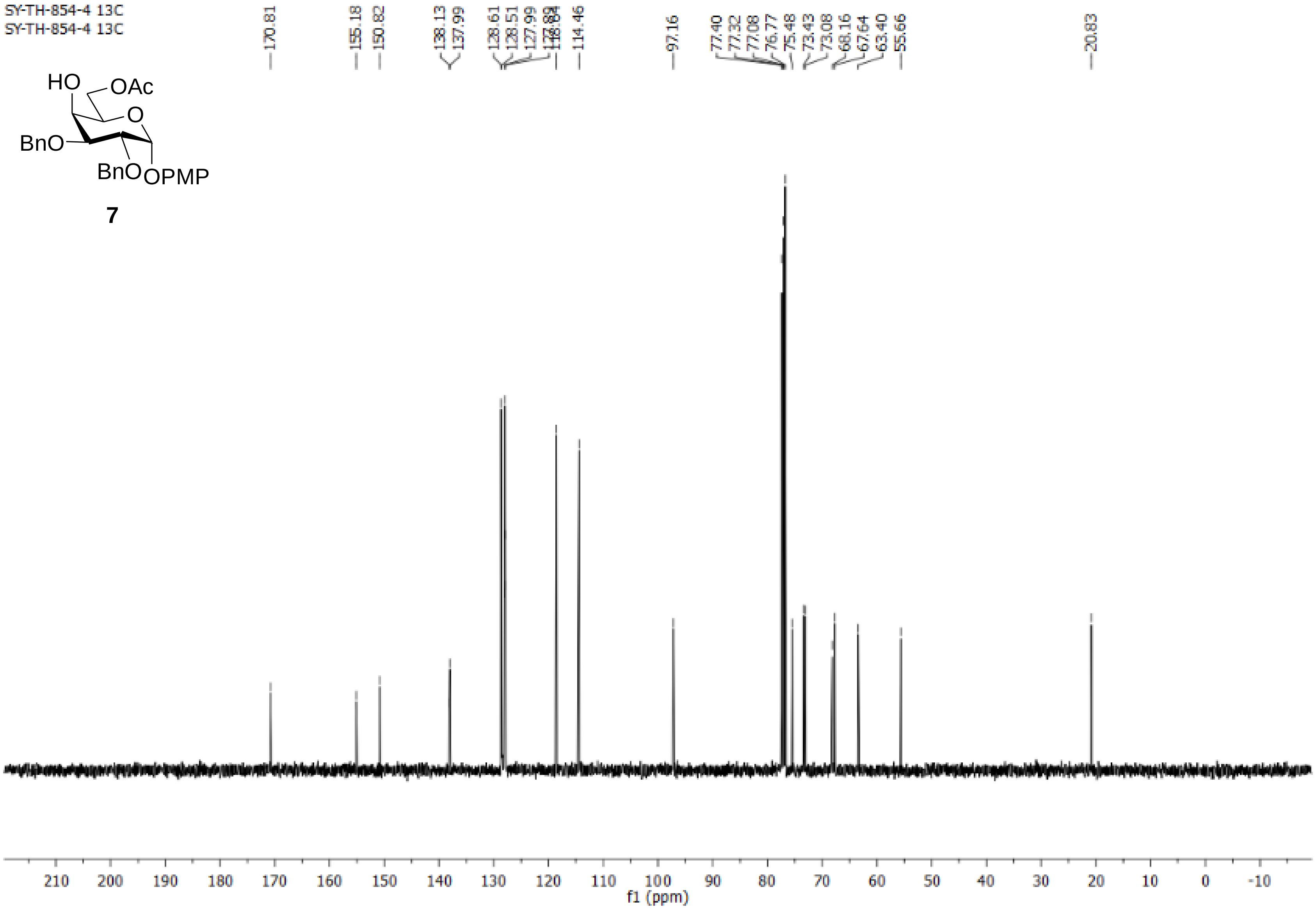

Figure S12: ^{13}C NMR spectrum (100 MHz, $CDCl_3$) of compound **7**.

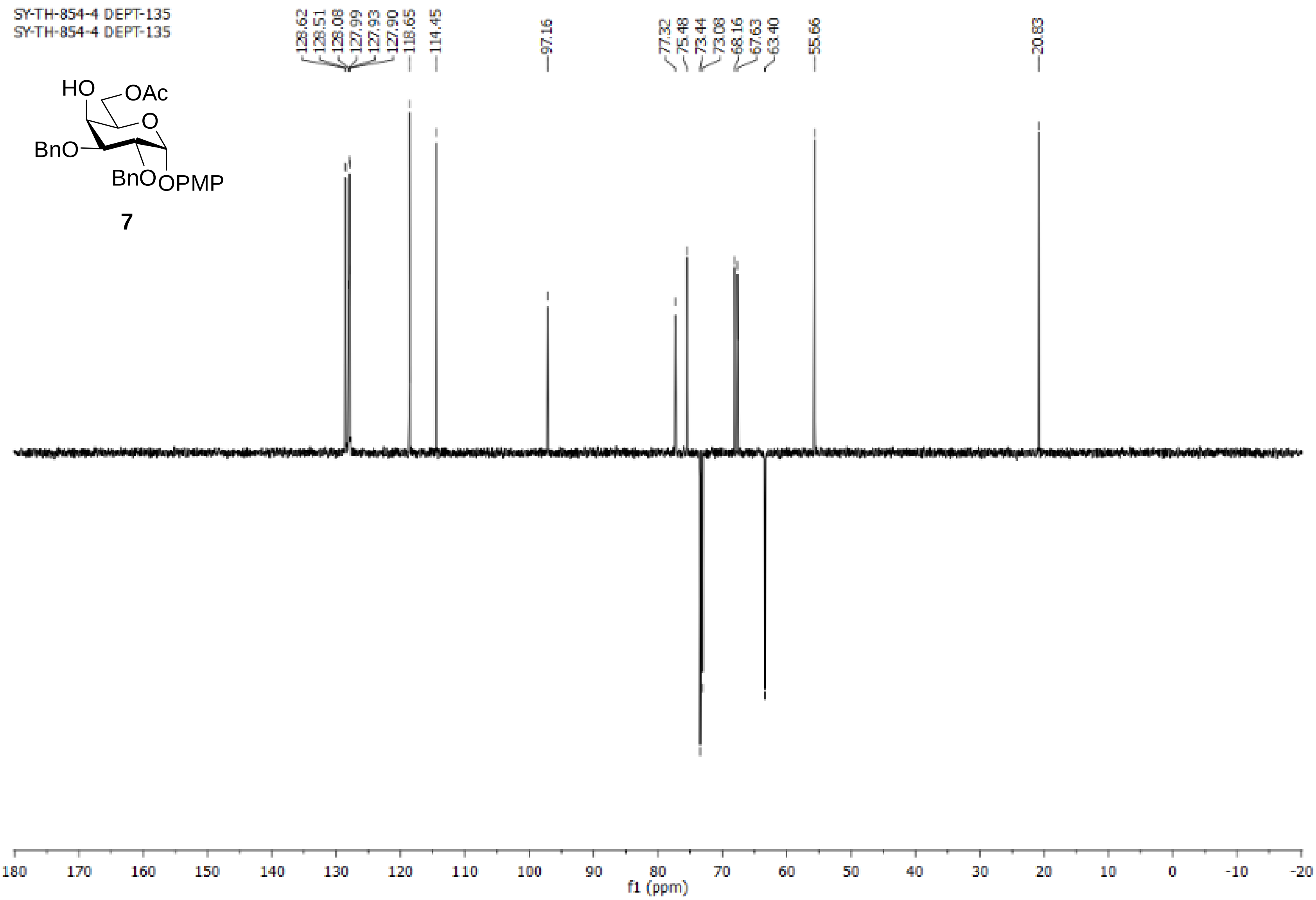

Figure S13: DEPT-135-NMR spectrum (100 MHz, $CDCl_3$) of compound **7**.

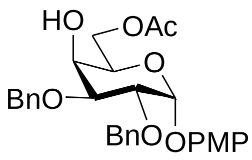

7

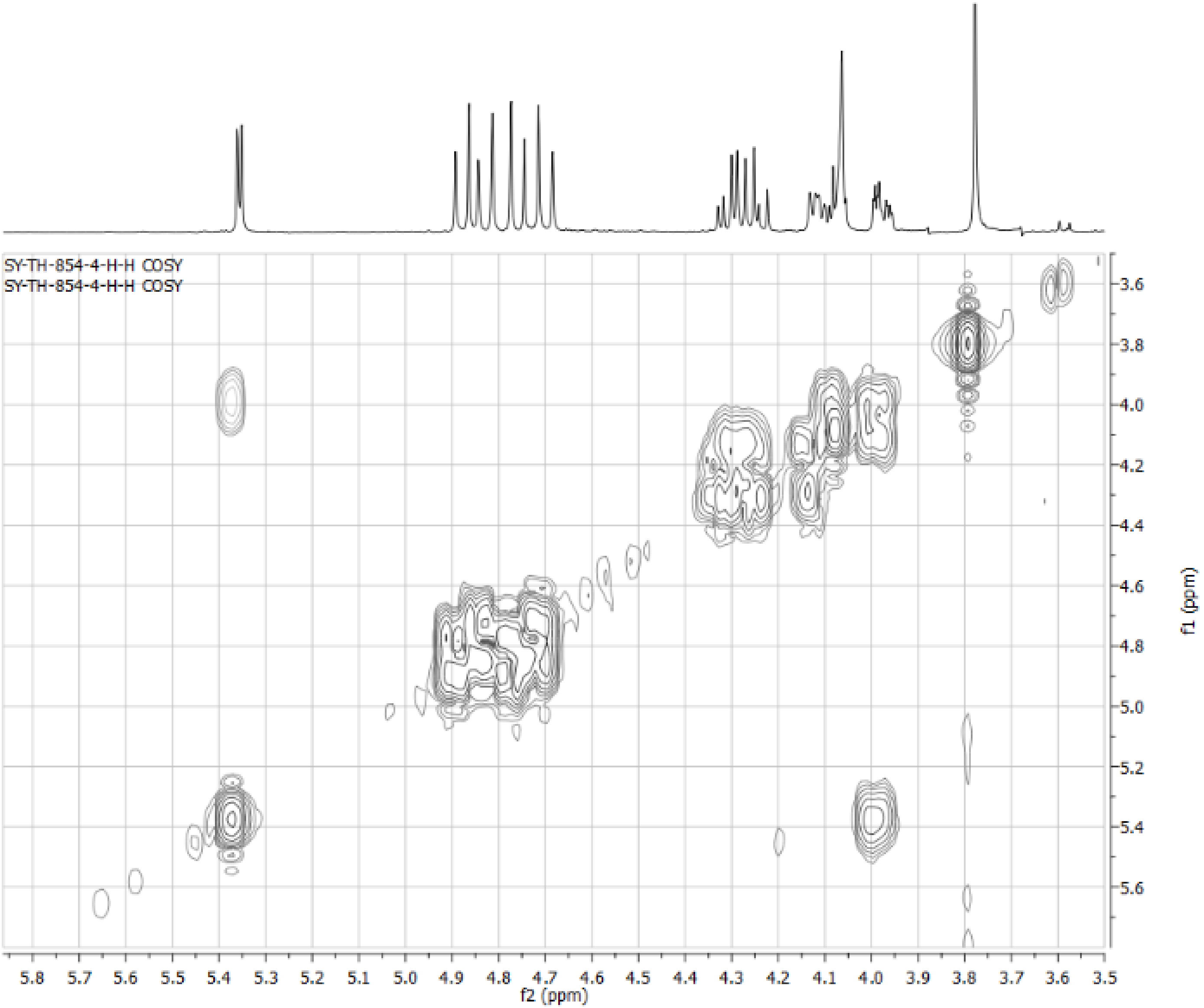

Figure S14: $^{1}H,^{1}H$-COSY-NMR spectrum (400 MHz, $CDCl_3$) of compound **7**.

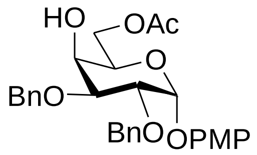

7

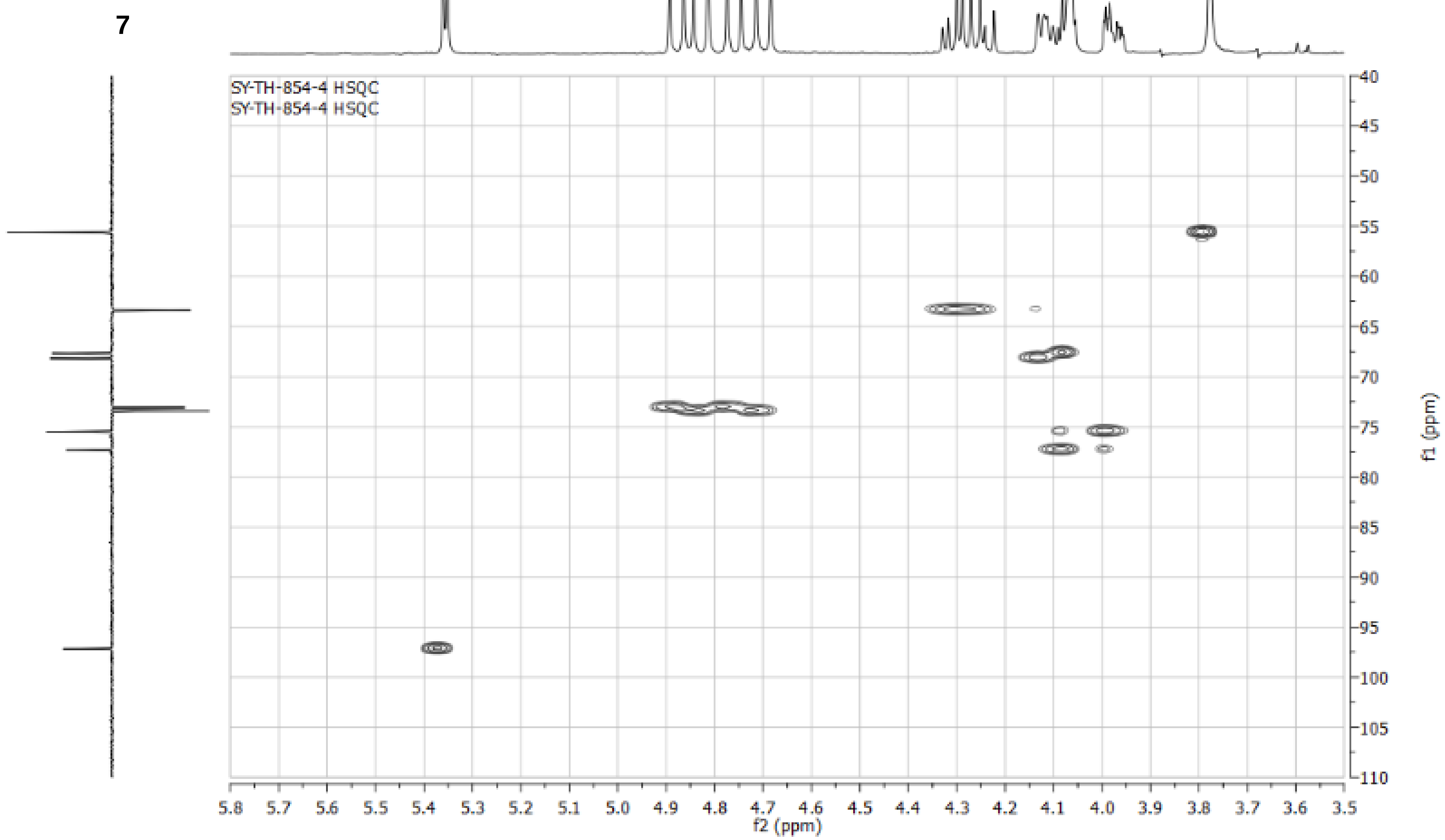

Figure S15: HSQC-NMR spectrum (400 MHz, $CDCl_3$) of compound **7**.

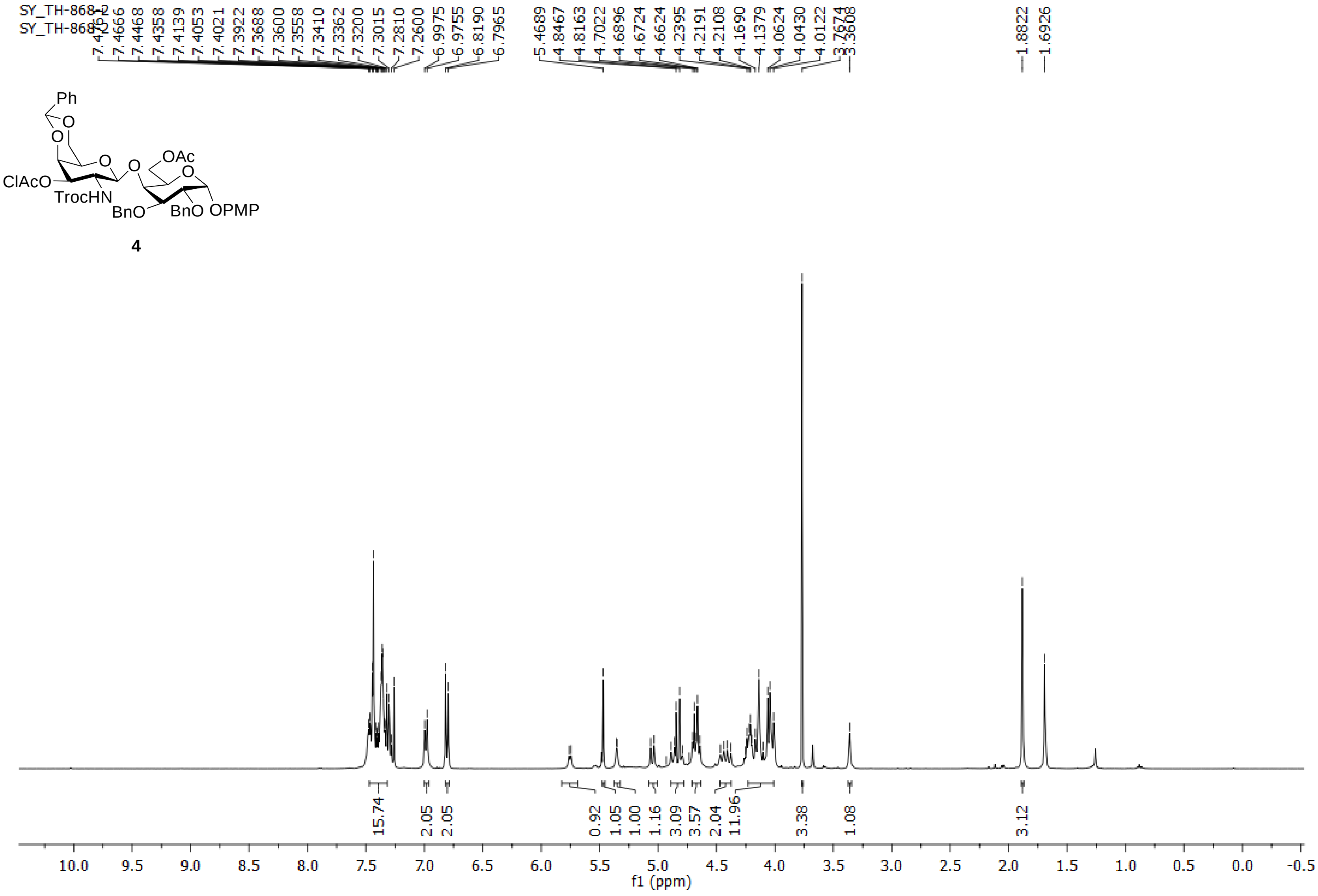

Figure S16: ^{1}H NMR spectrum (400 MHz, $CDCl_3$) of compound **4**.

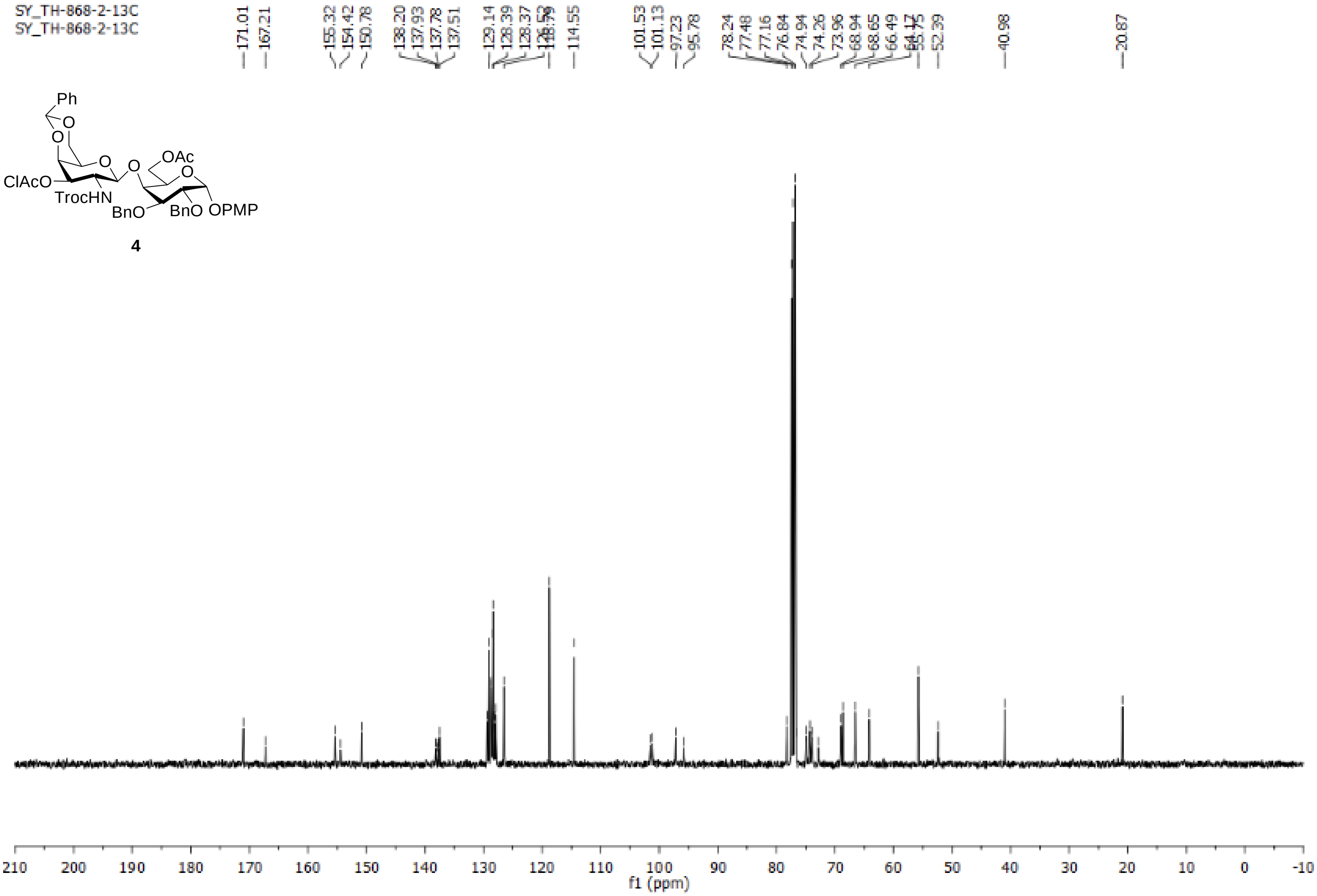

Figure S17: ^{13}C NMR spectrum (100 MHz, $CDCl_3$) of compound **4**.

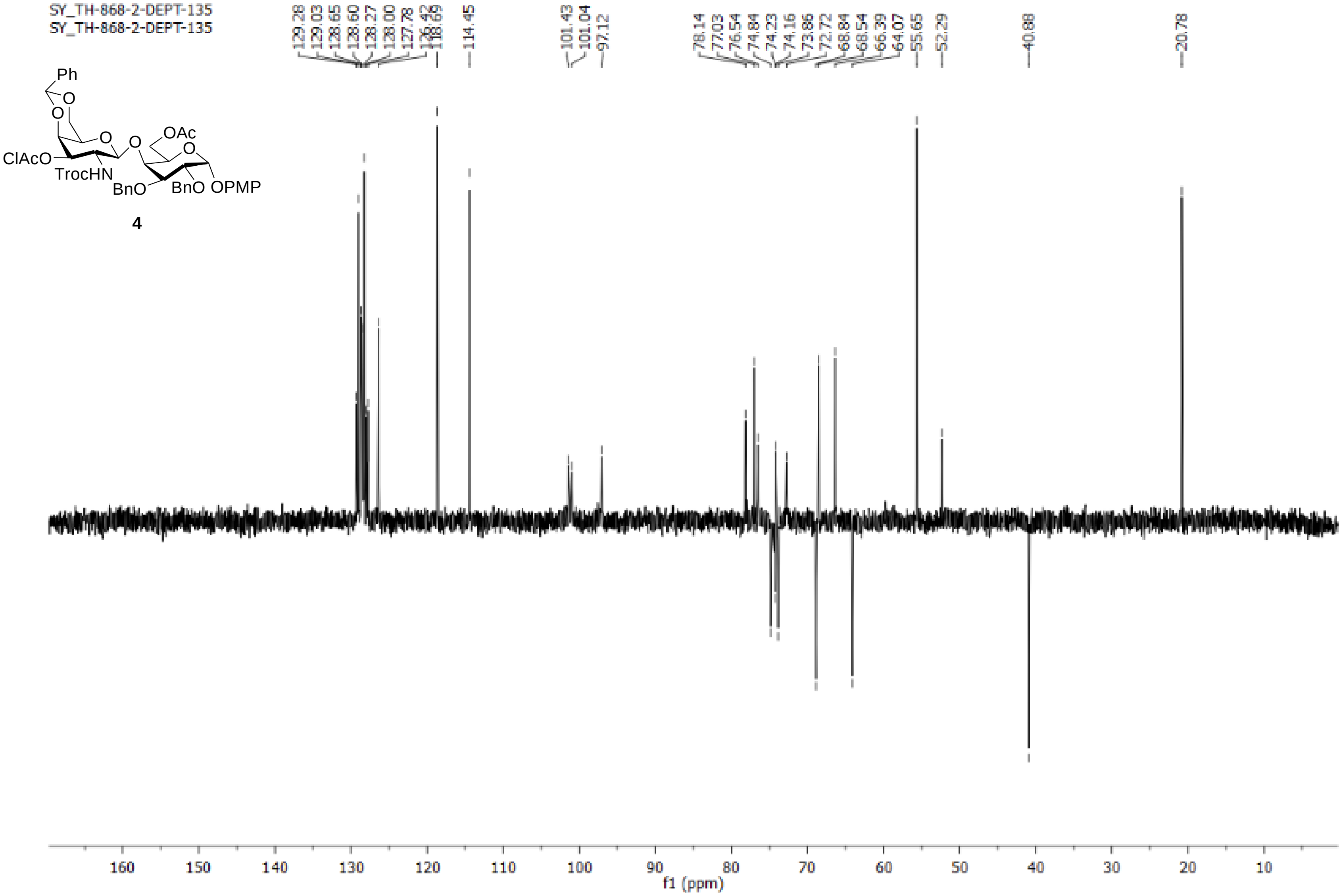

Figure S18: DEPT-135-NMR spectrum (100 MHz, $CDCl_3$) of compound **4**.

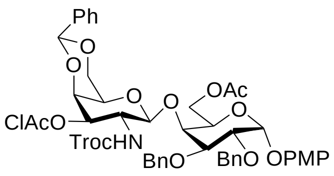

4

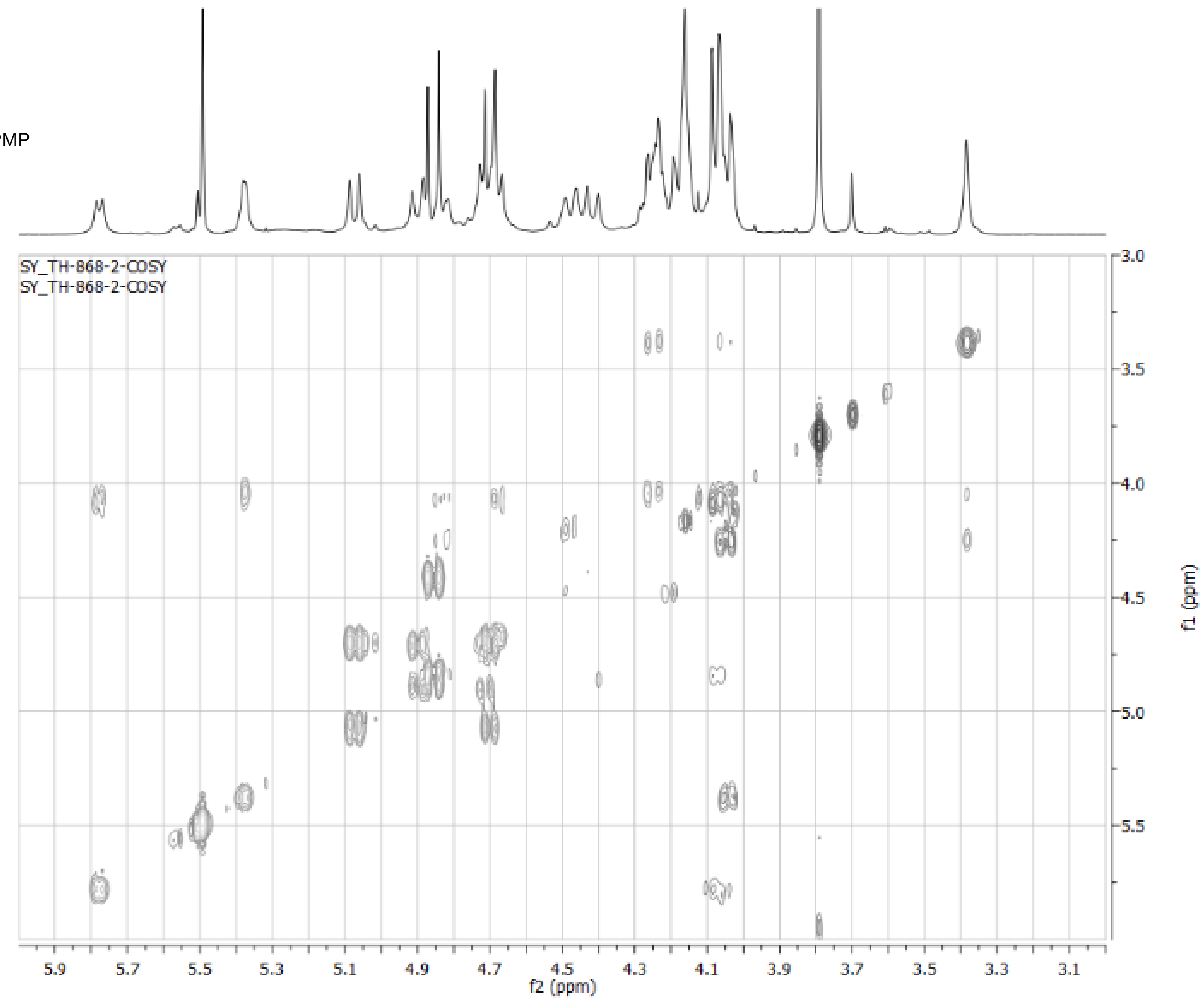

Figure S19: ^{1}H,^{1}H-COSY-NMR spectrum (400 MHz, $CDCl_3$) of compound **4**.

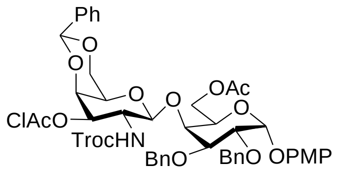

4

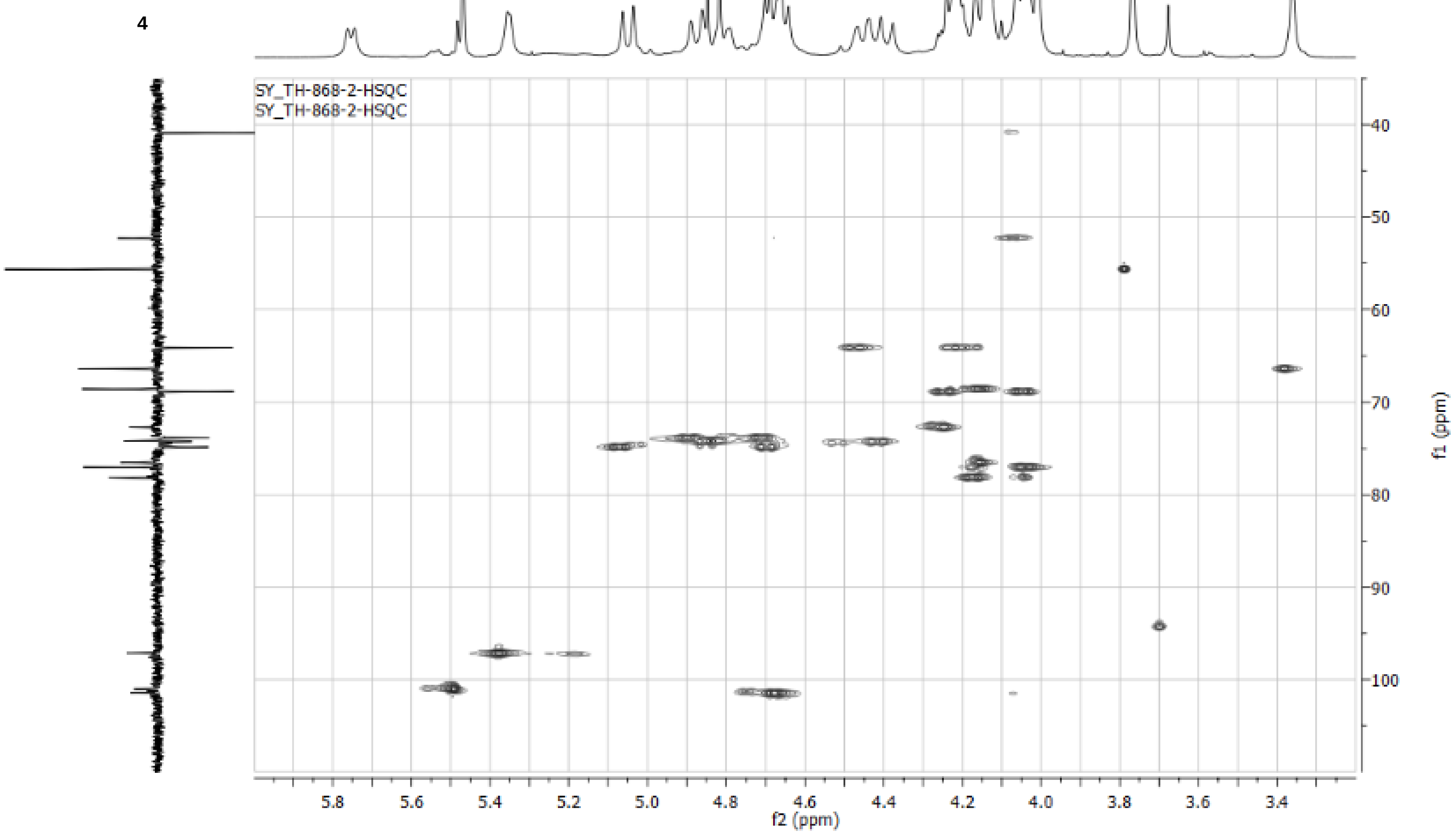

Figure S20: HSQC-NMR spectrum (400 MHz, $CDCl_3$) of compound **4**.

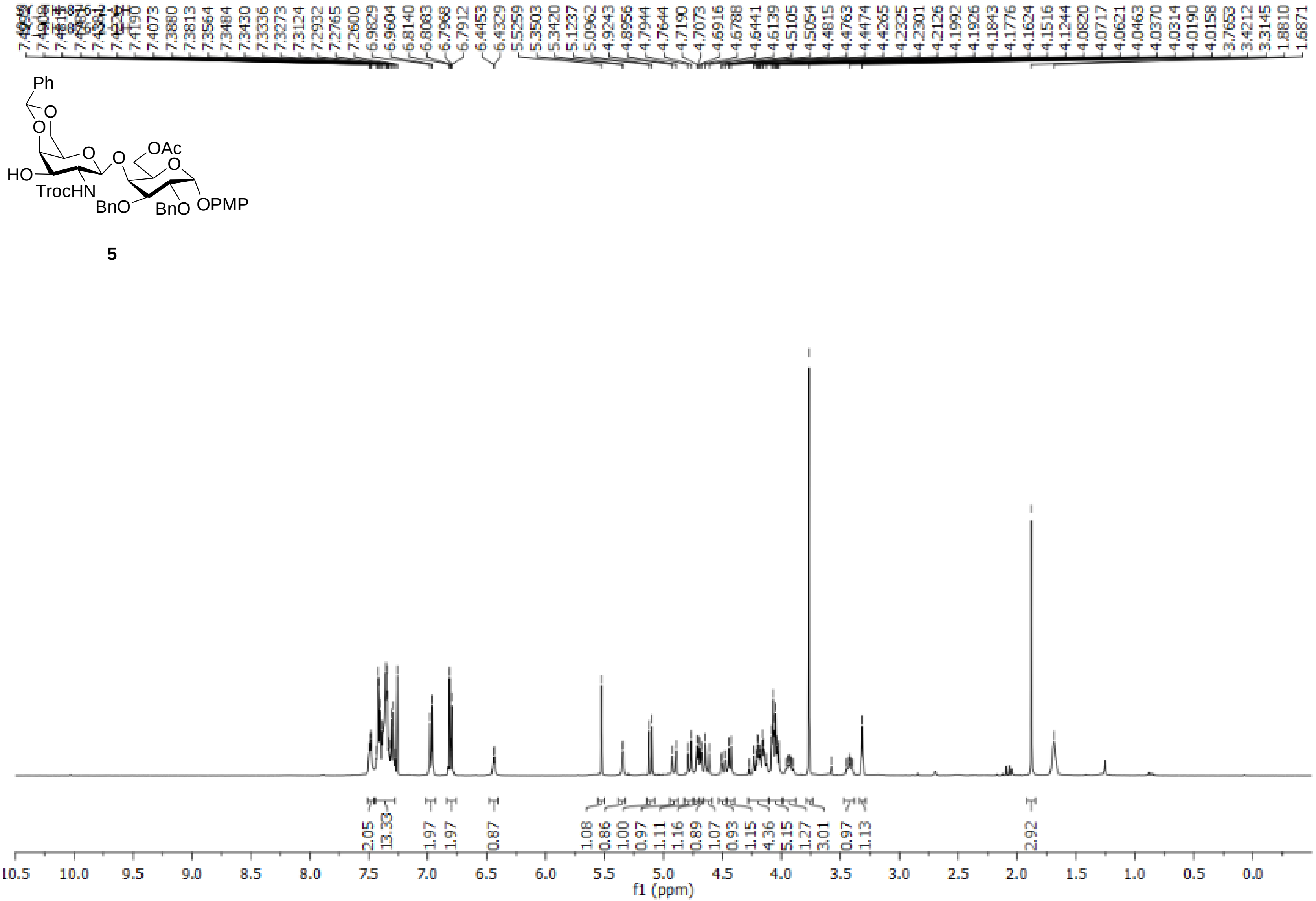

Figure S21: ^{1}H NMR spectrum (400 MHz, $CDCl_3$) of compound **5**.

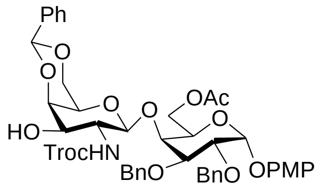

5

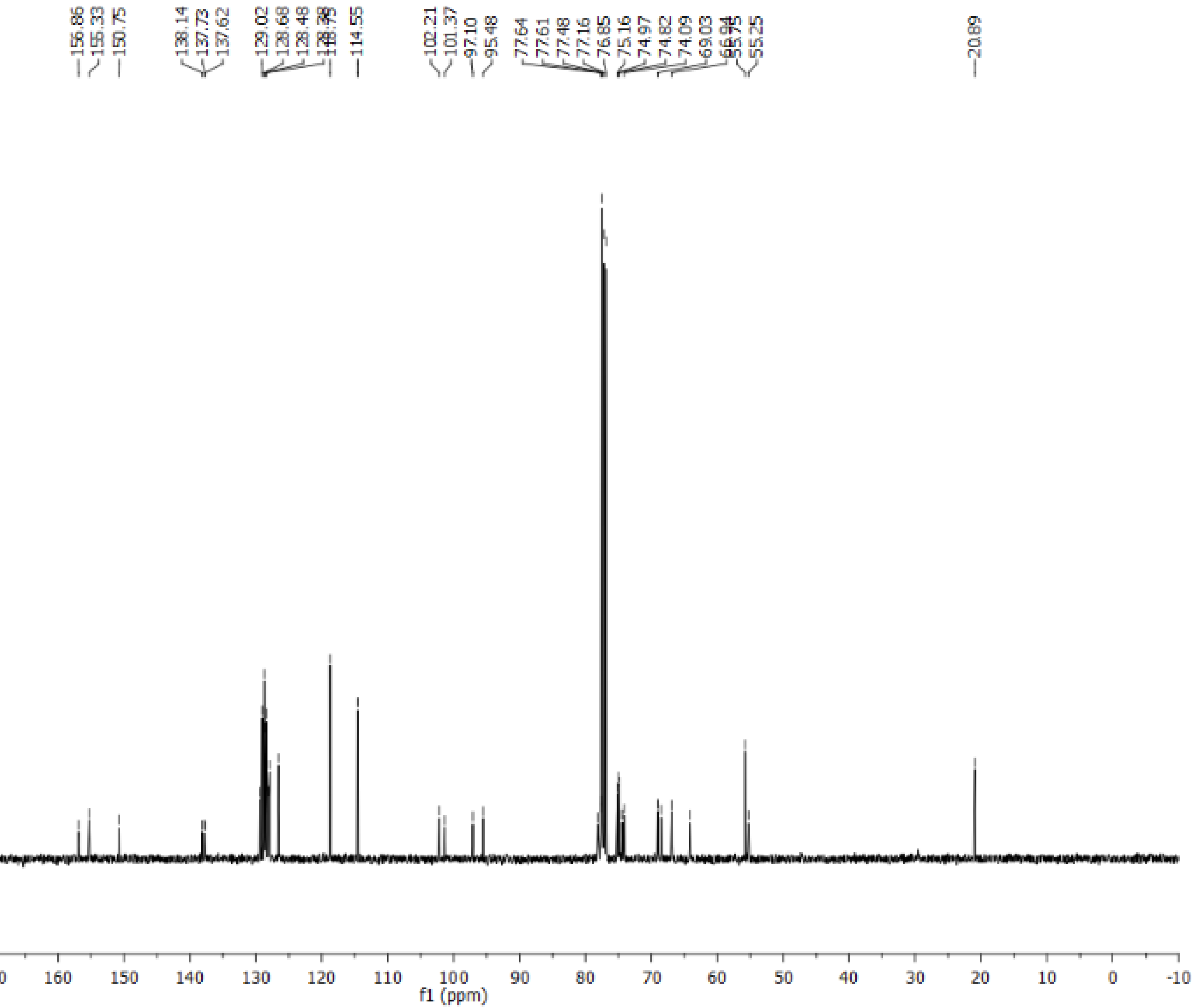

Figure S22: ^{13}C NMR spectrum (100 MHz, $CDCl_3$) of compound **5**.

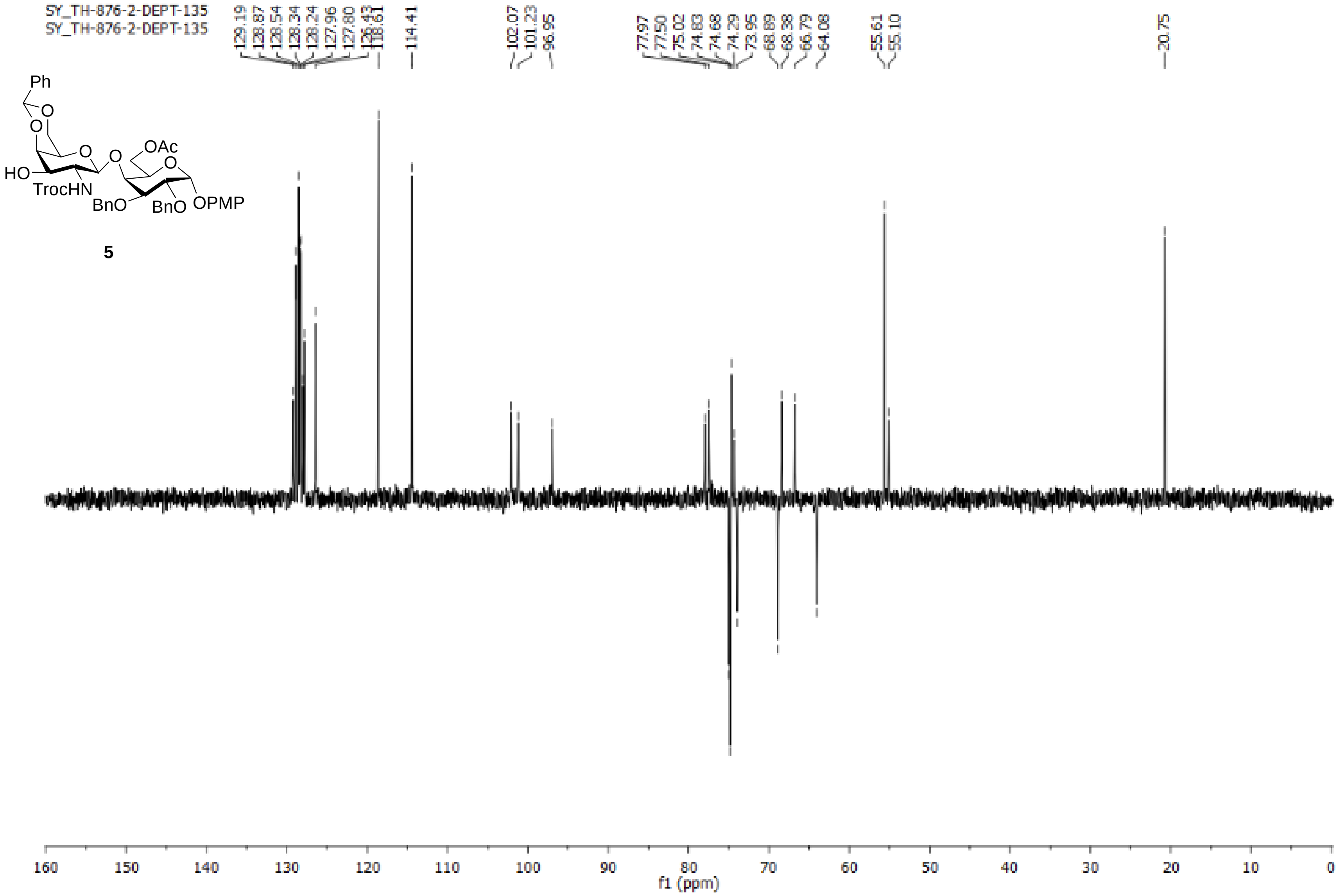

Figure S23: DEPT-135-NMR spectrum (100 MHz, $CDCl_3$) of compound **5**.

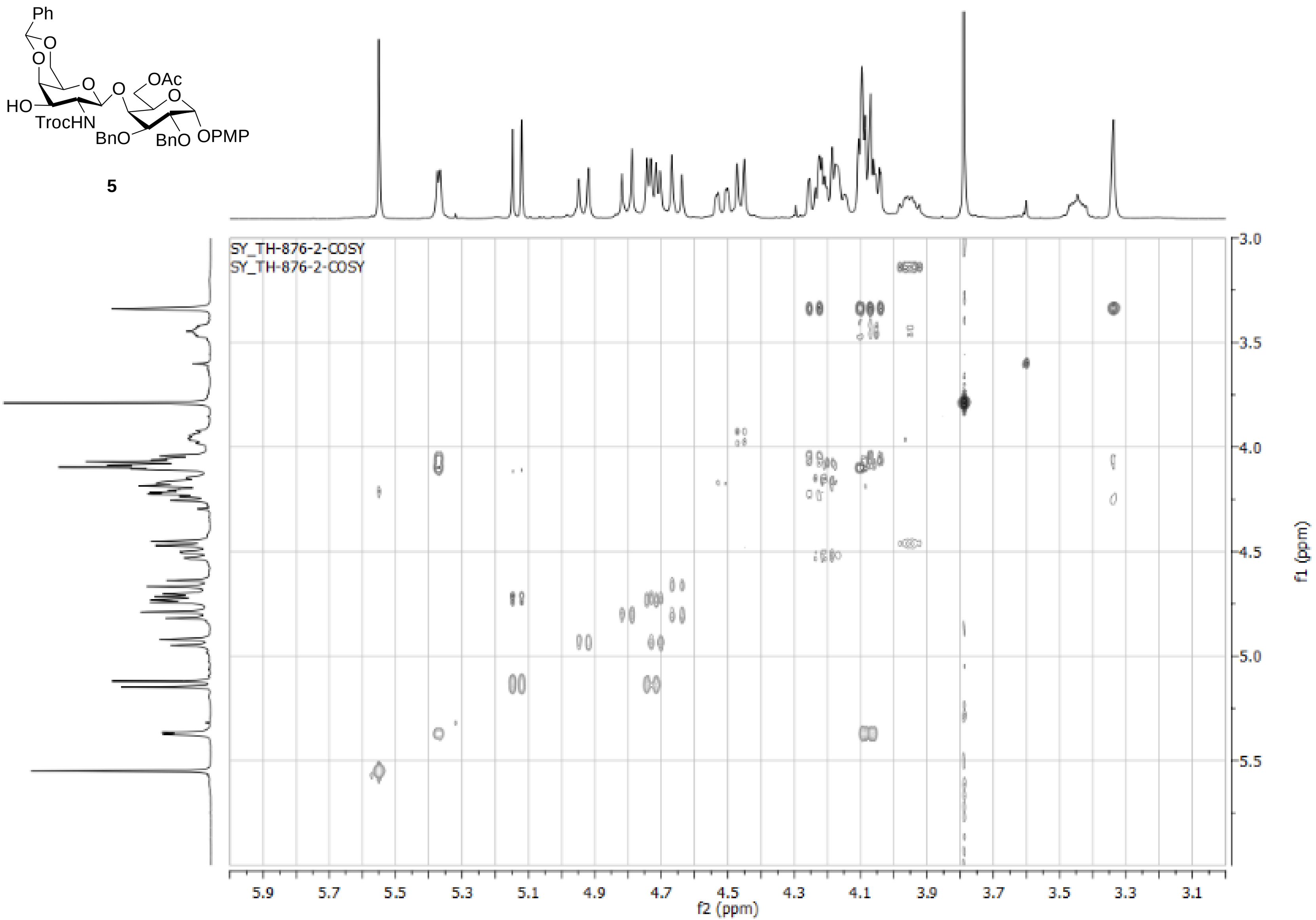

Figure S24: $^{1}H,^{1}H$-COSY-NMR spectrum (400 MHz, $CDCl_3$) of compound **5**.

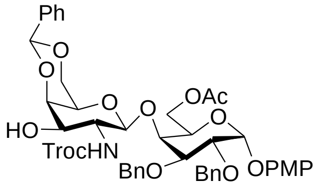

5

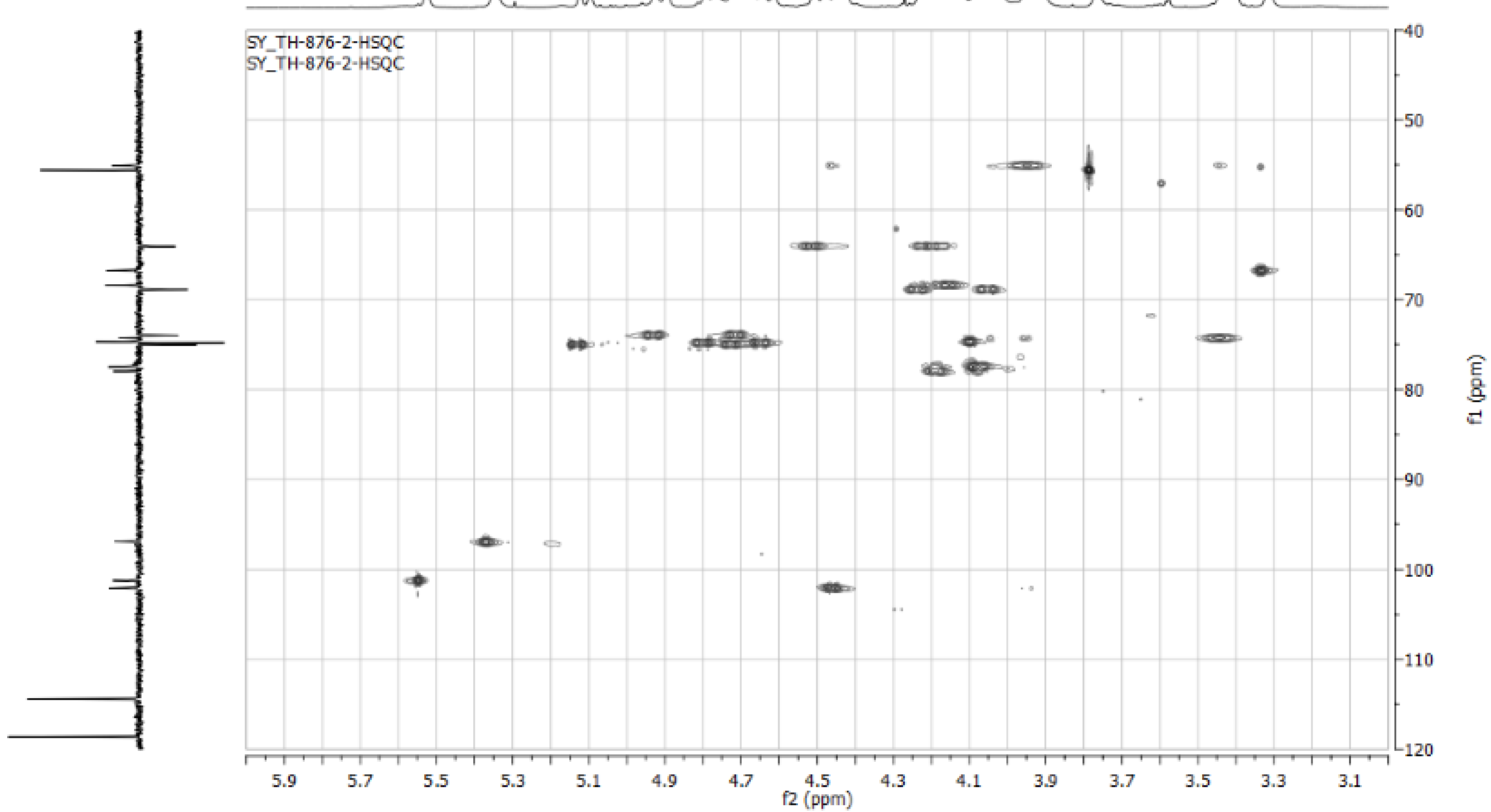

Figure S25: HSQC-NMR spectrum (400 MHz, $CDCl_3$) of compound **5**.

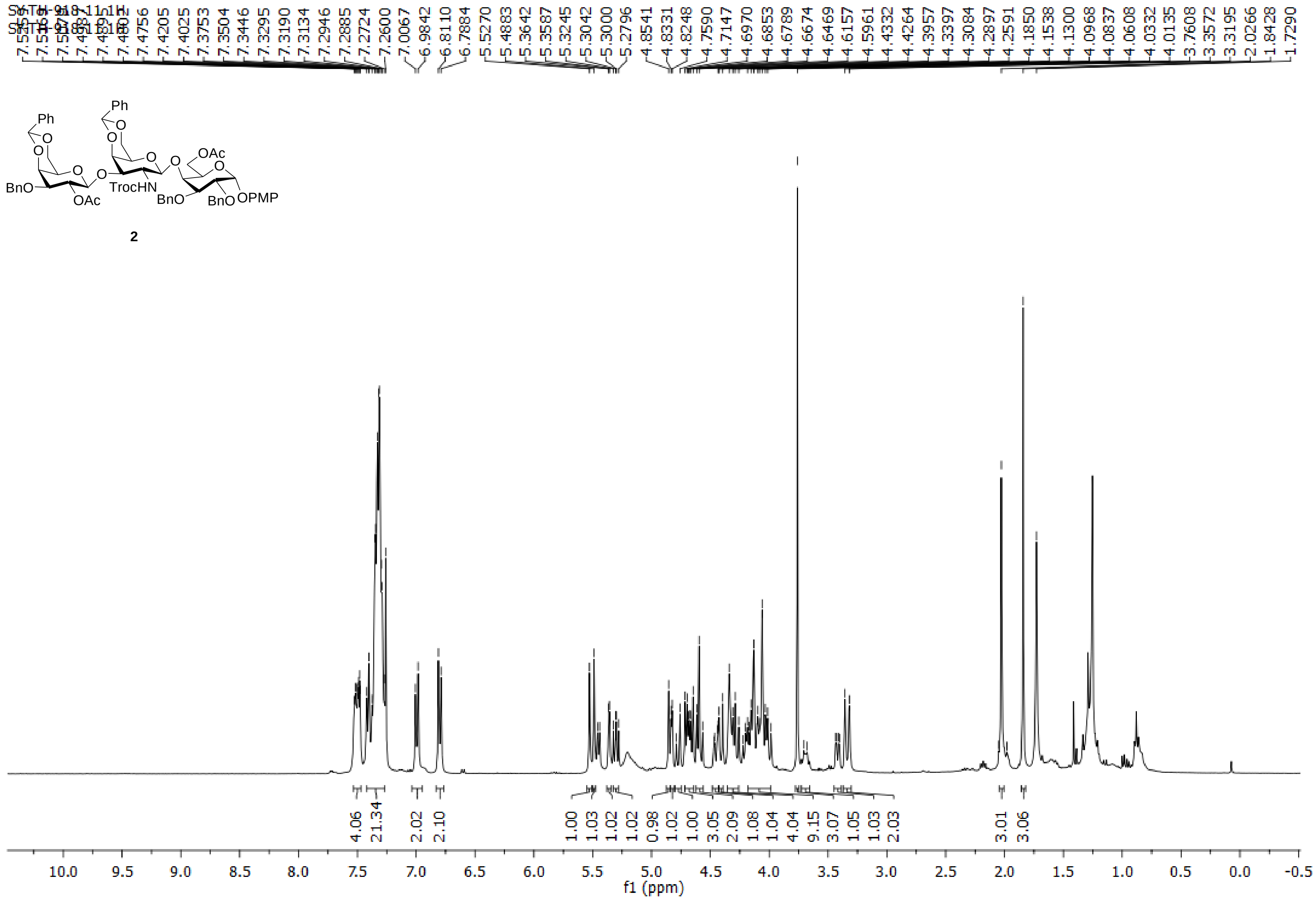

Figure S26: ^{1}H NMR spectrum (400 MHz, $CDCl_3$) of compound **2**.

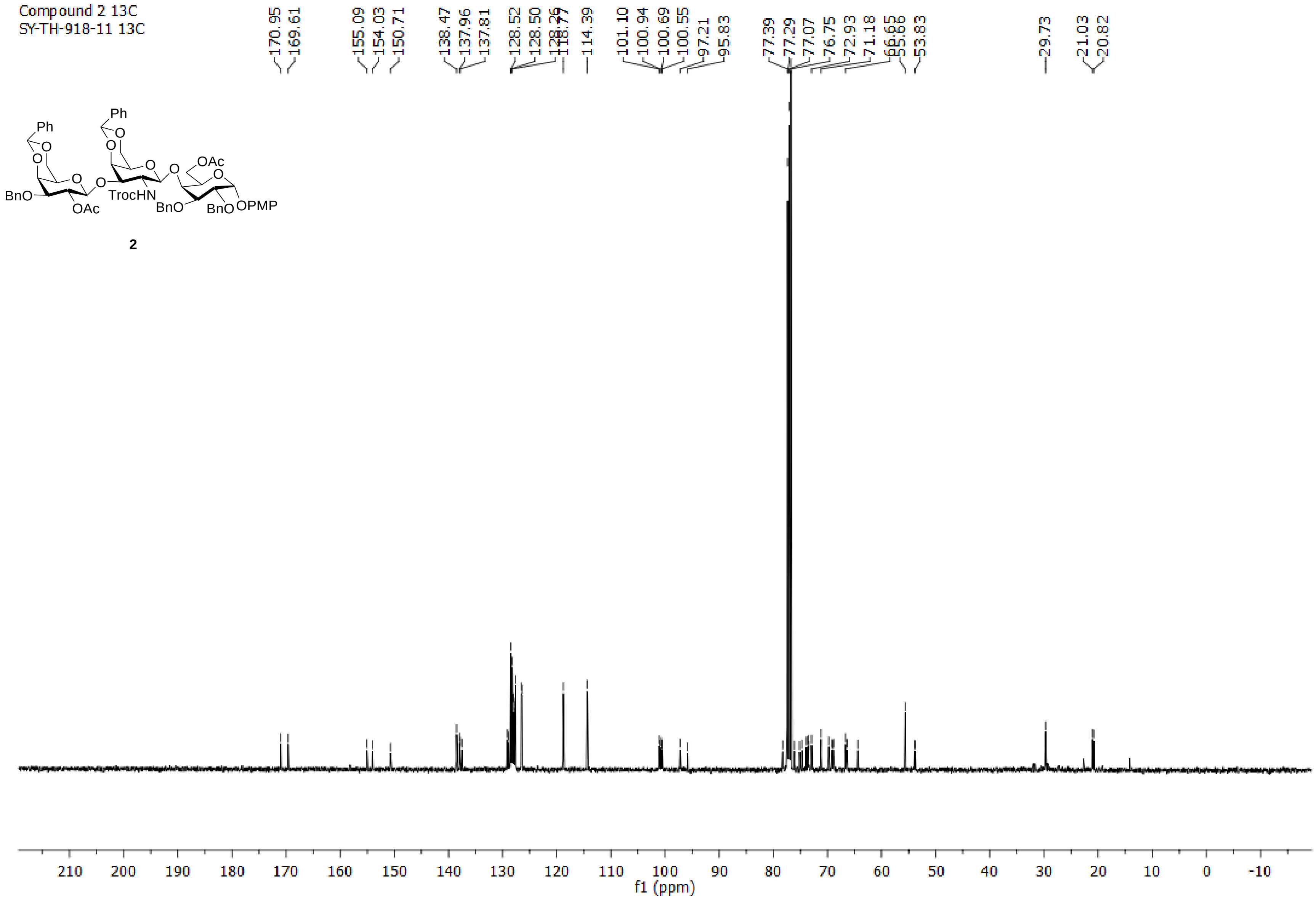

Figure S27: ^{13}C NMR spectrum (100 MHz, $CDCl_3$) of compound **2**.

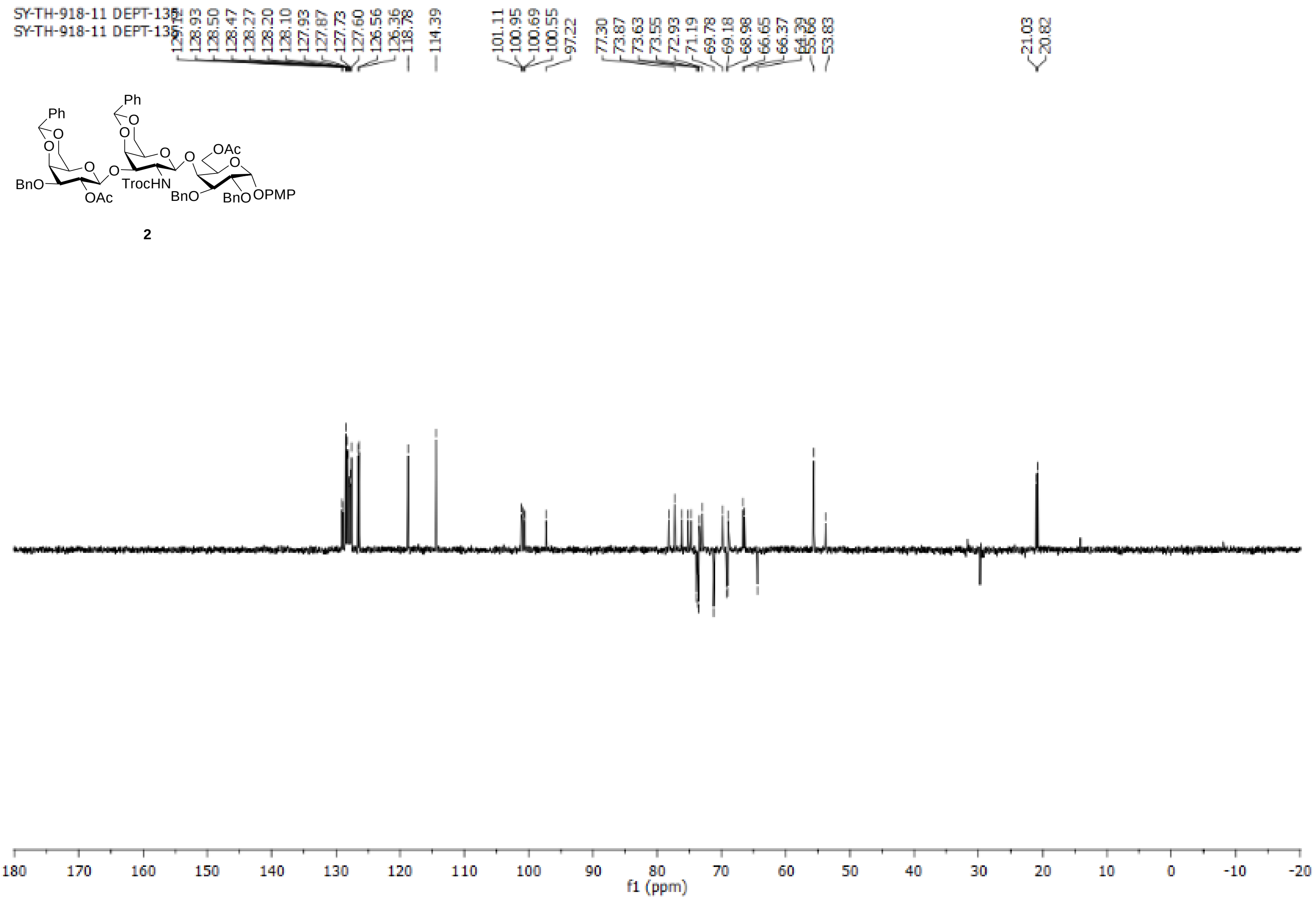

Figure S28: DEPT-135-NMR spectrum (100 MHz, $CDCl_3$) of compound **2**.

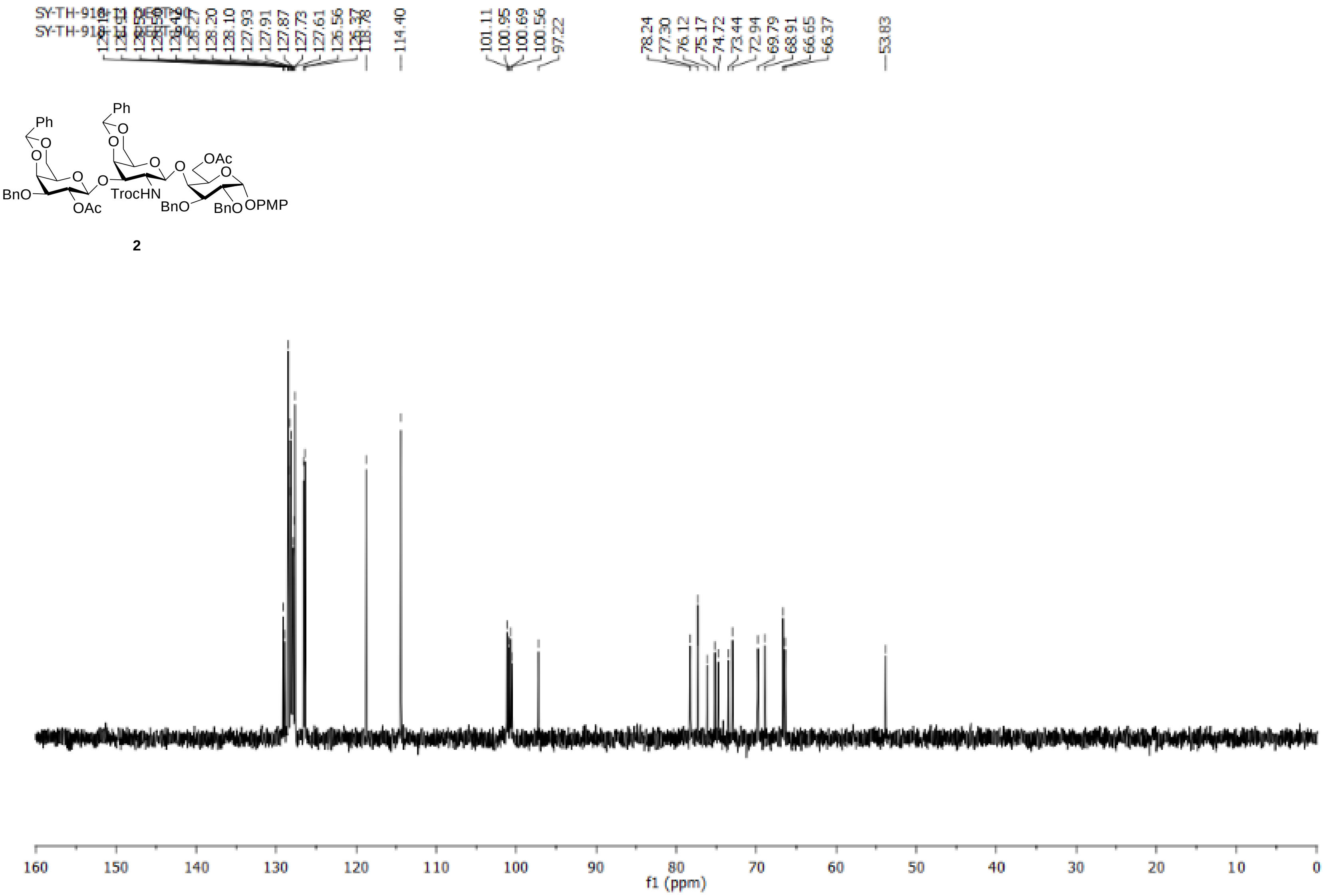

Figure S29: DEPT-90-NMR spectrum (100 MHz, $CDCl_3$) of compound **2**.

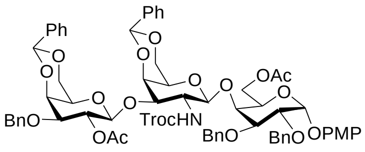

2

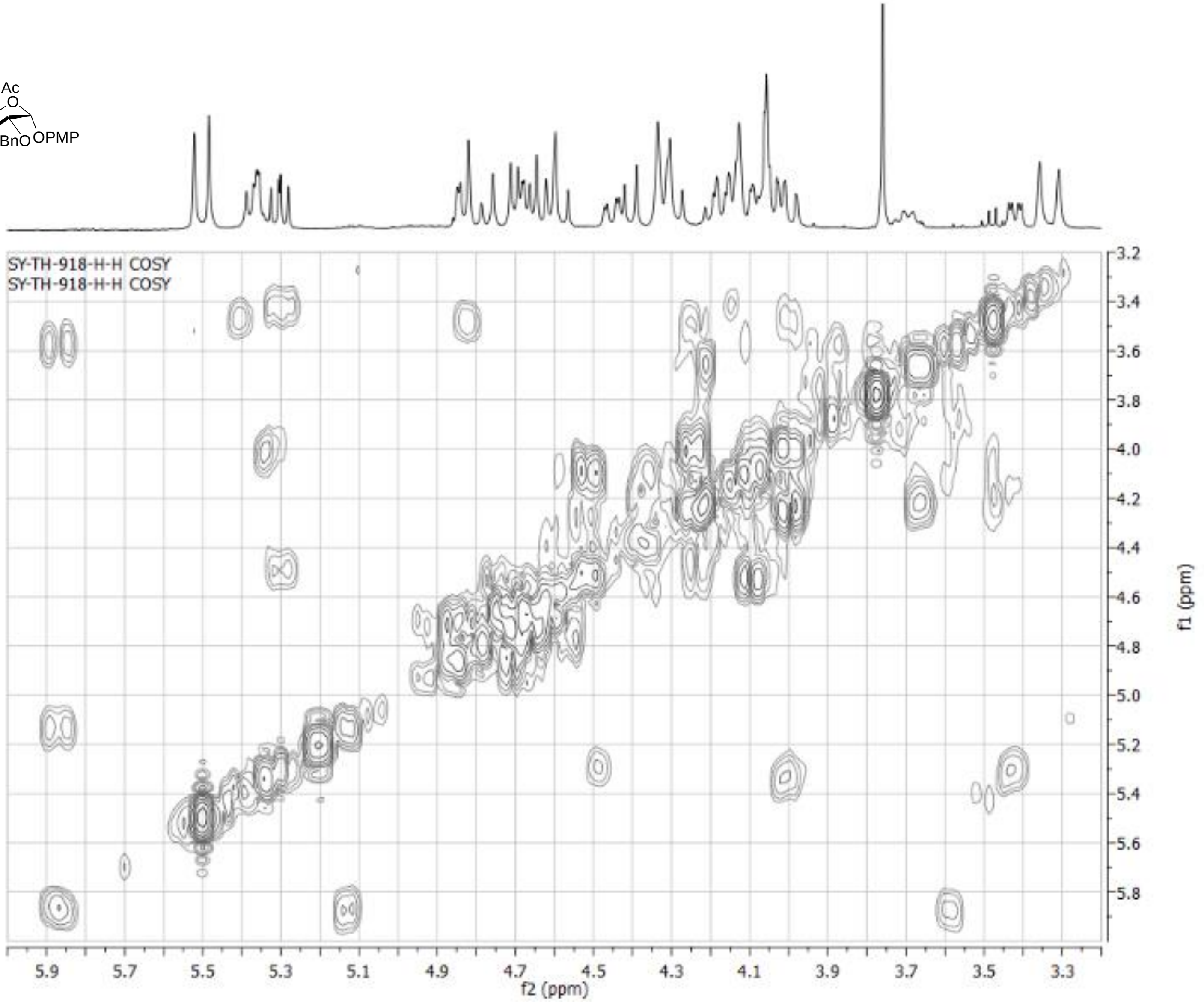

Figure S30: ^{1}H,^{1}H-COSY-NMR spectrum (400 MHz, $CDCl_3$) of compound **2**.

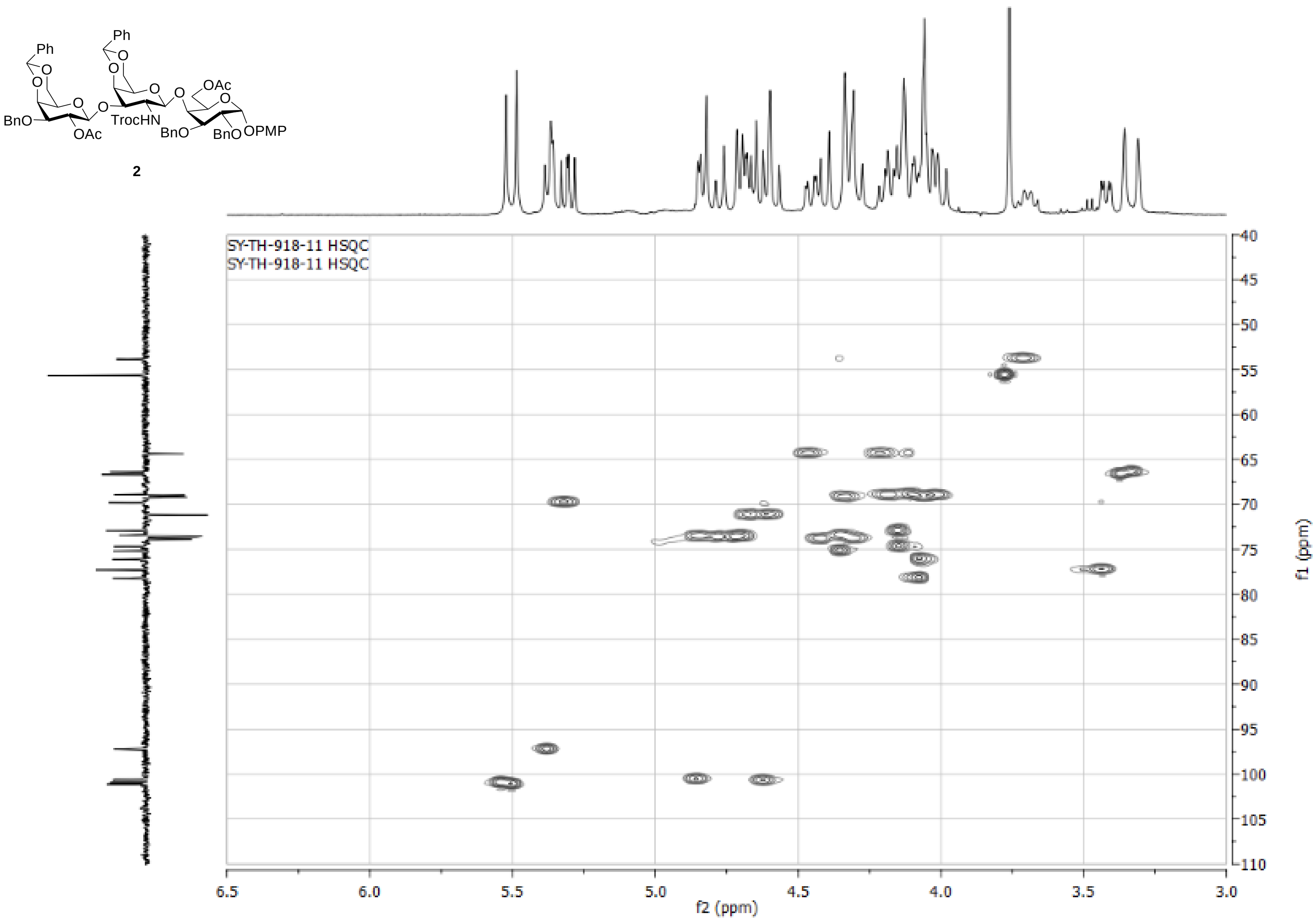

Figure S31: HSQC-NMR spectrum (400 MHz, $CDCl_3$) of compound **2**.

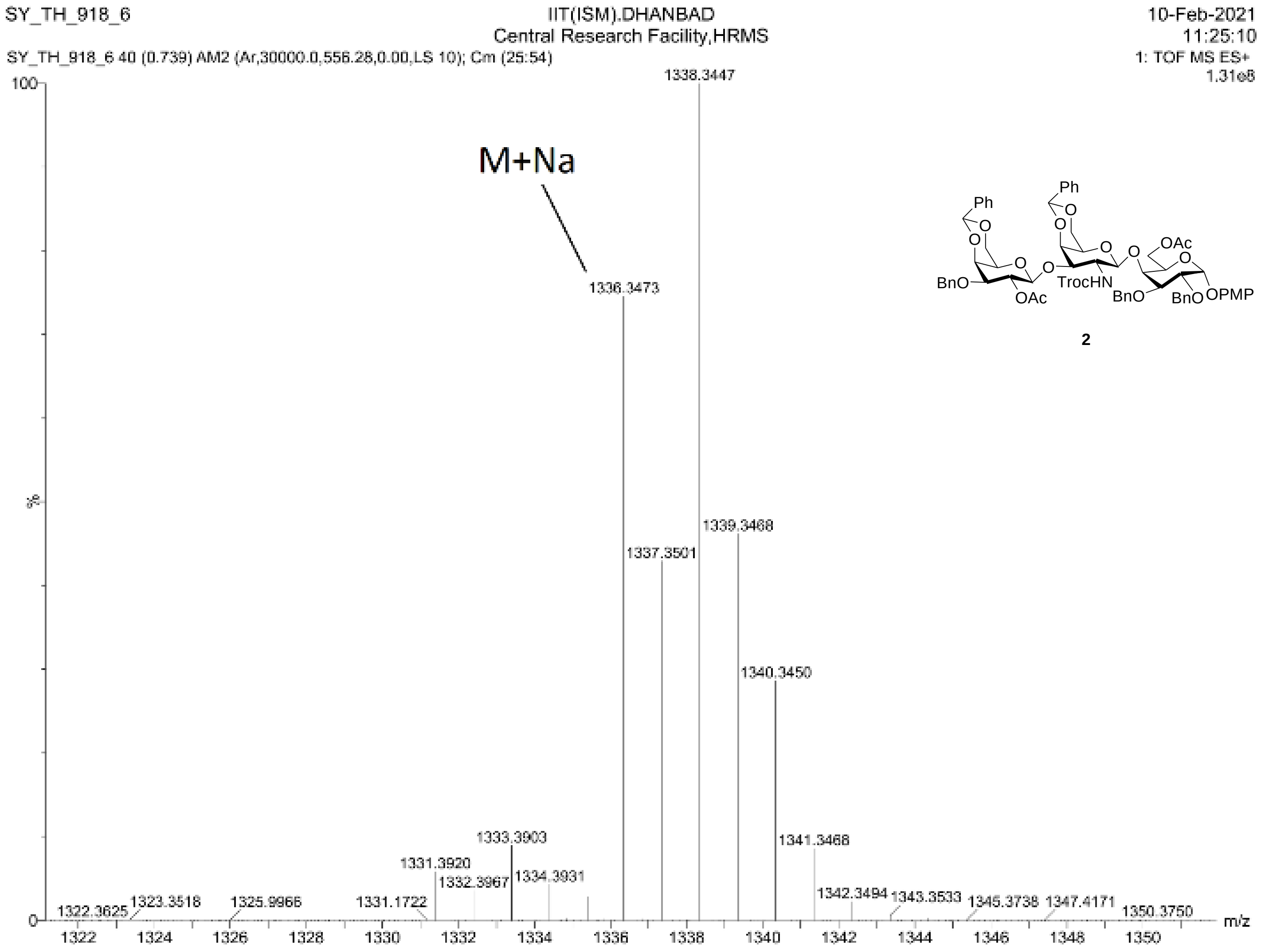

Figure S32: HRMS spectrum of compound **2**.

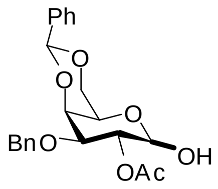

11

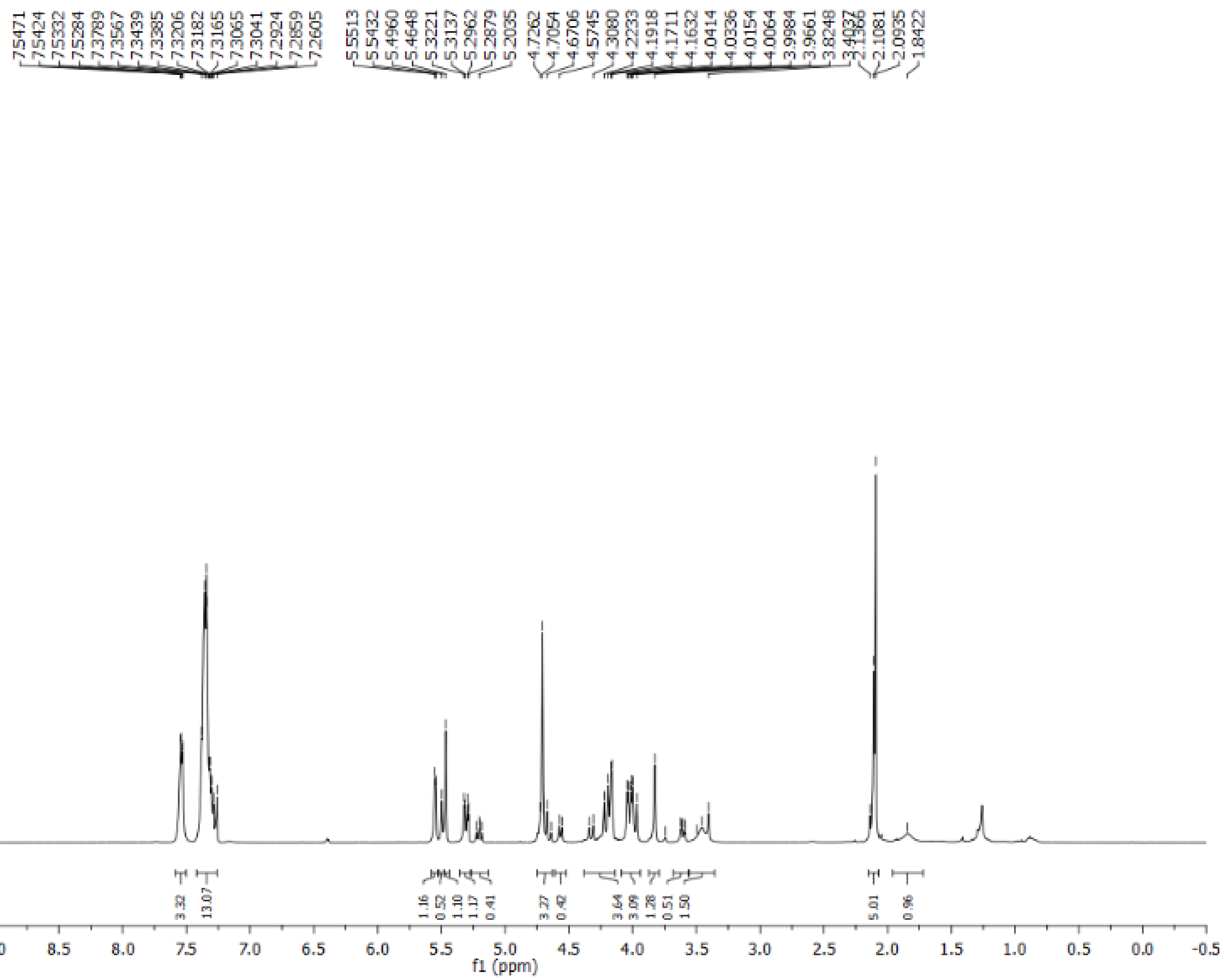

Figure S33: ^{1}H NMR spectrum (400 MHz, $CDCl_3$) of compound **11**.

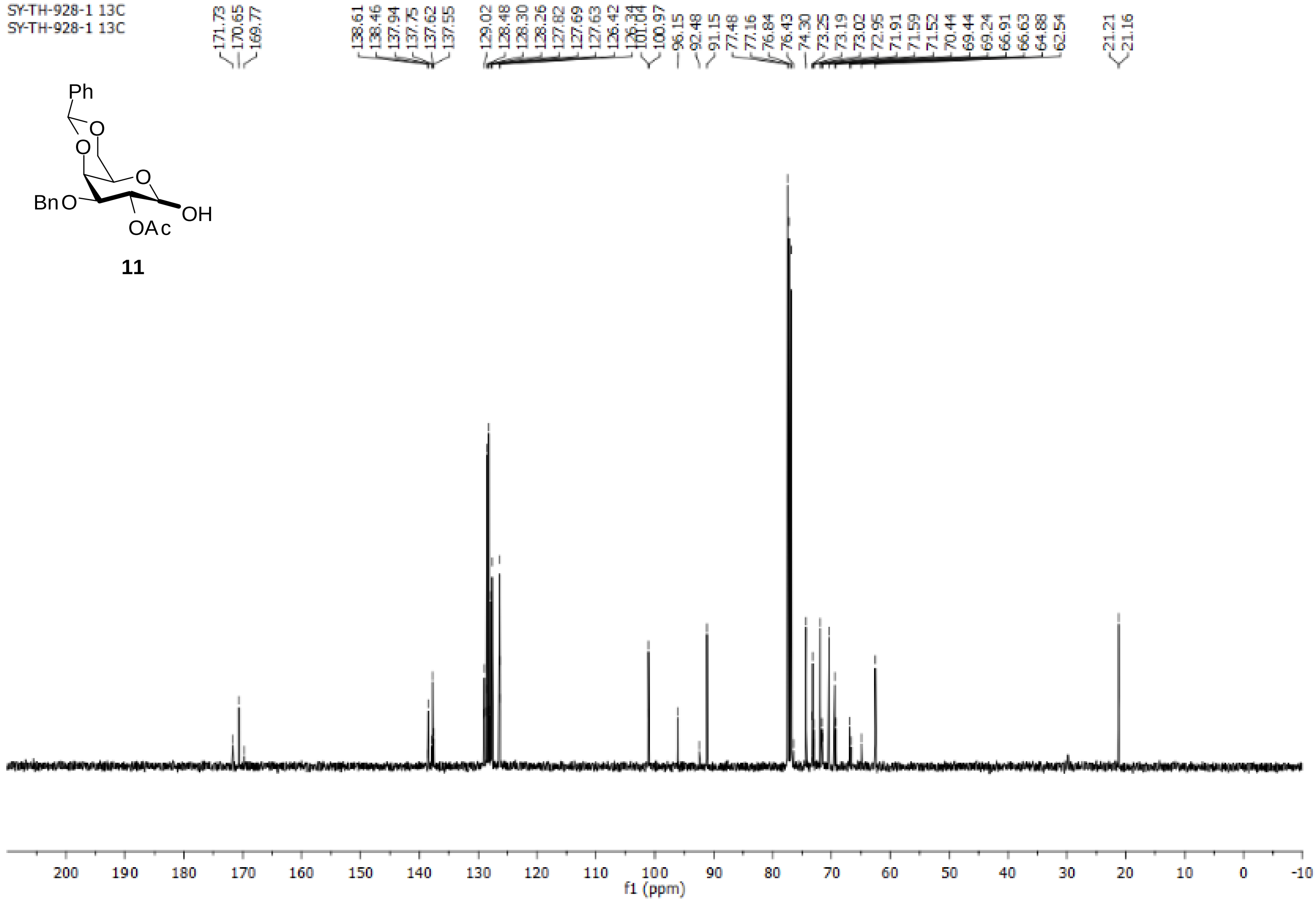

Figure S34: ^{13}C NMR spectrum (100 MHz, $CDCl_3$) of compound **11**.

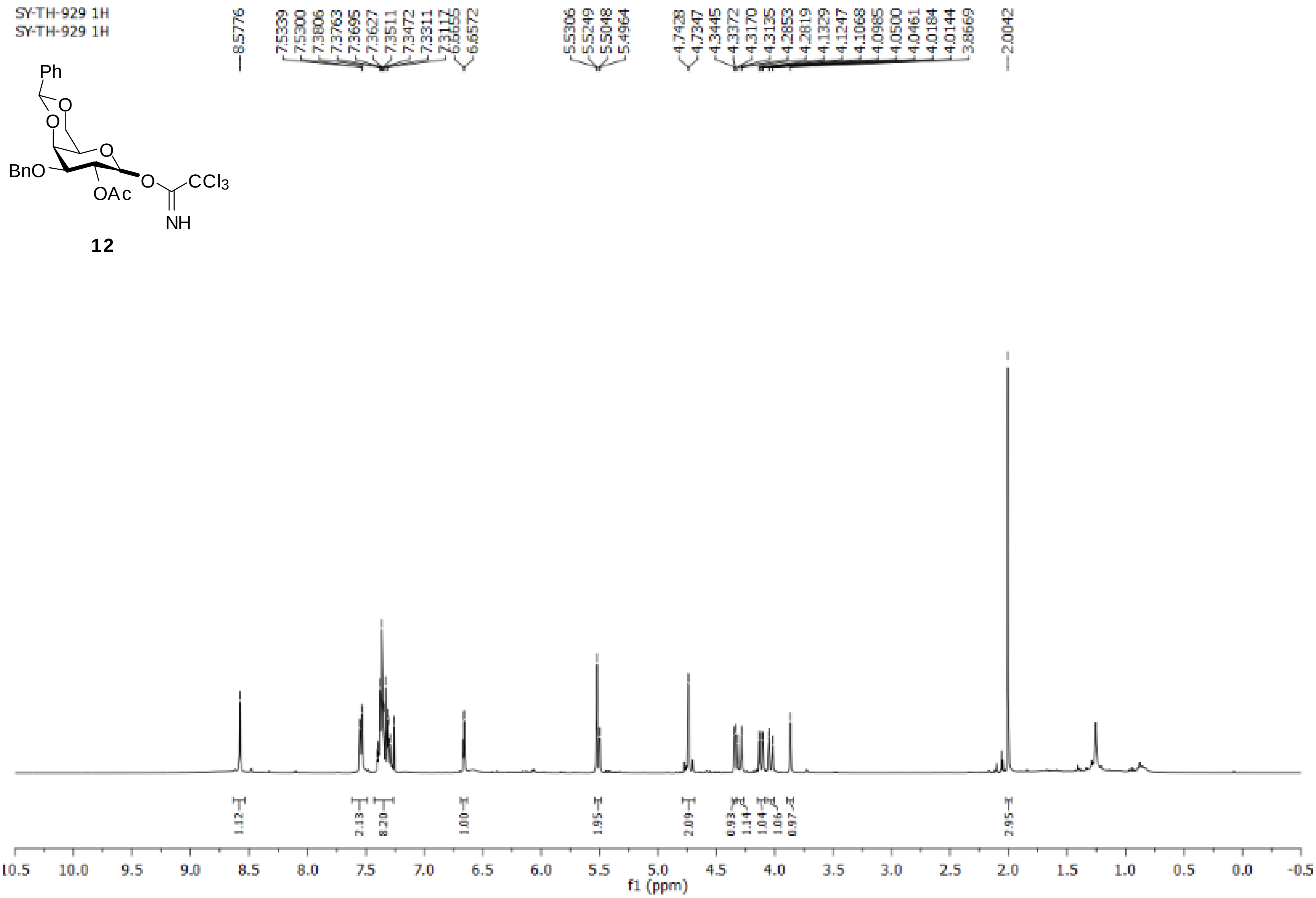

Figure S35: ^{1}H NMR spectrum (400 MHz, $CDCl_3$) of compound **12**.

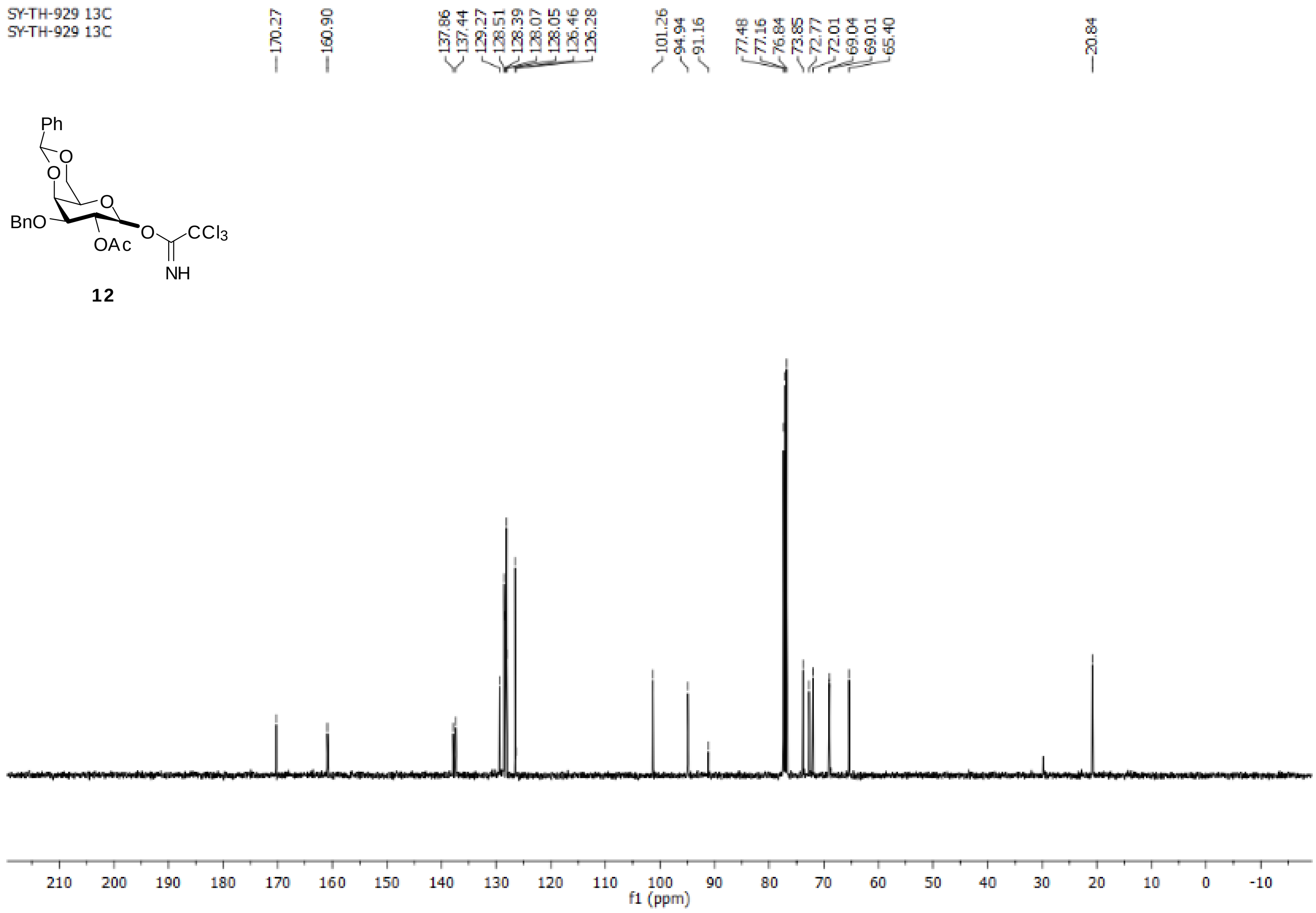

12

Figure S36: ^{13}C NMR spectrum (100 MHz, $CDCl_3$) of compound **12**.

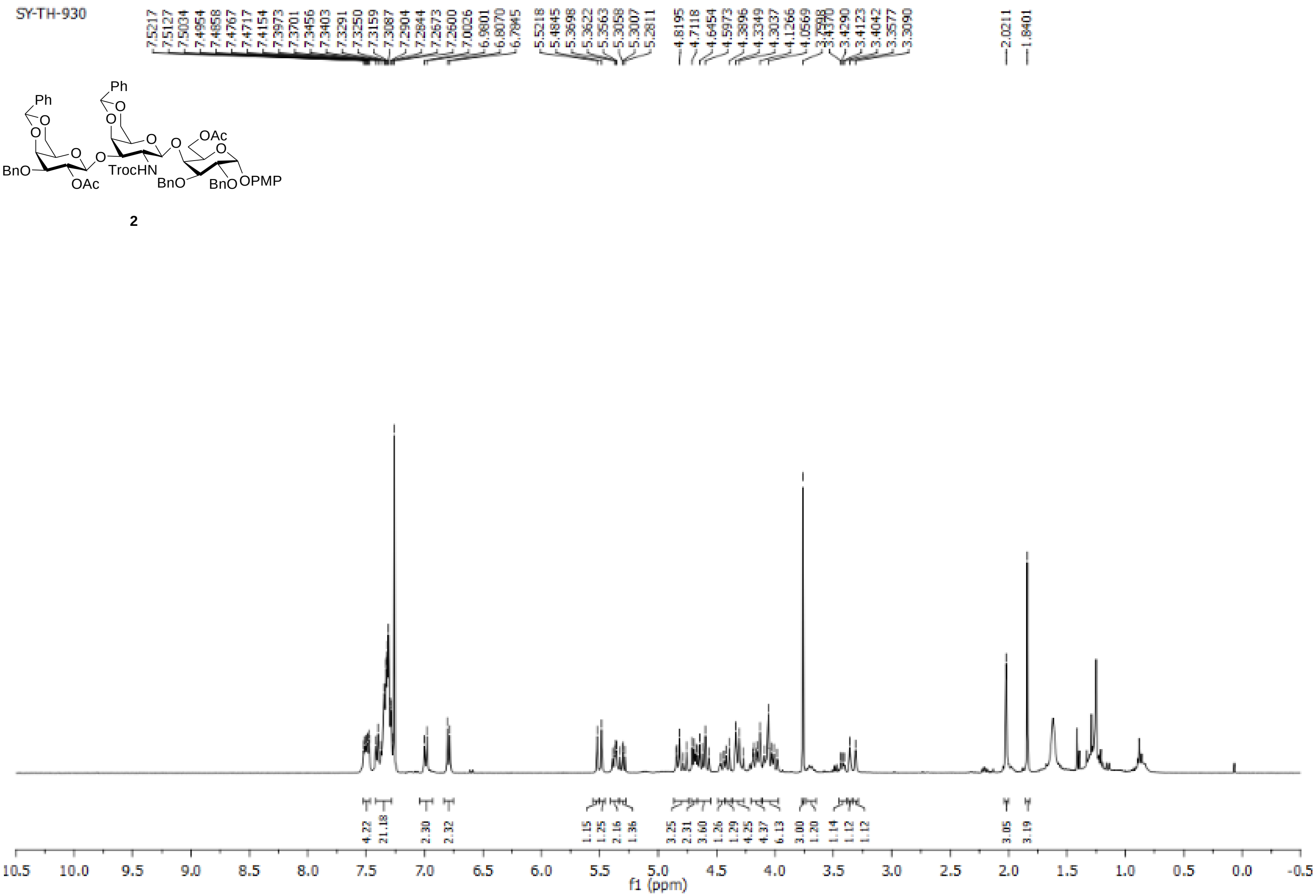

Figure S37: ${}^{1}H$ NMR spectrum (400 MHz, $CDCl_3$) of compound **2**.

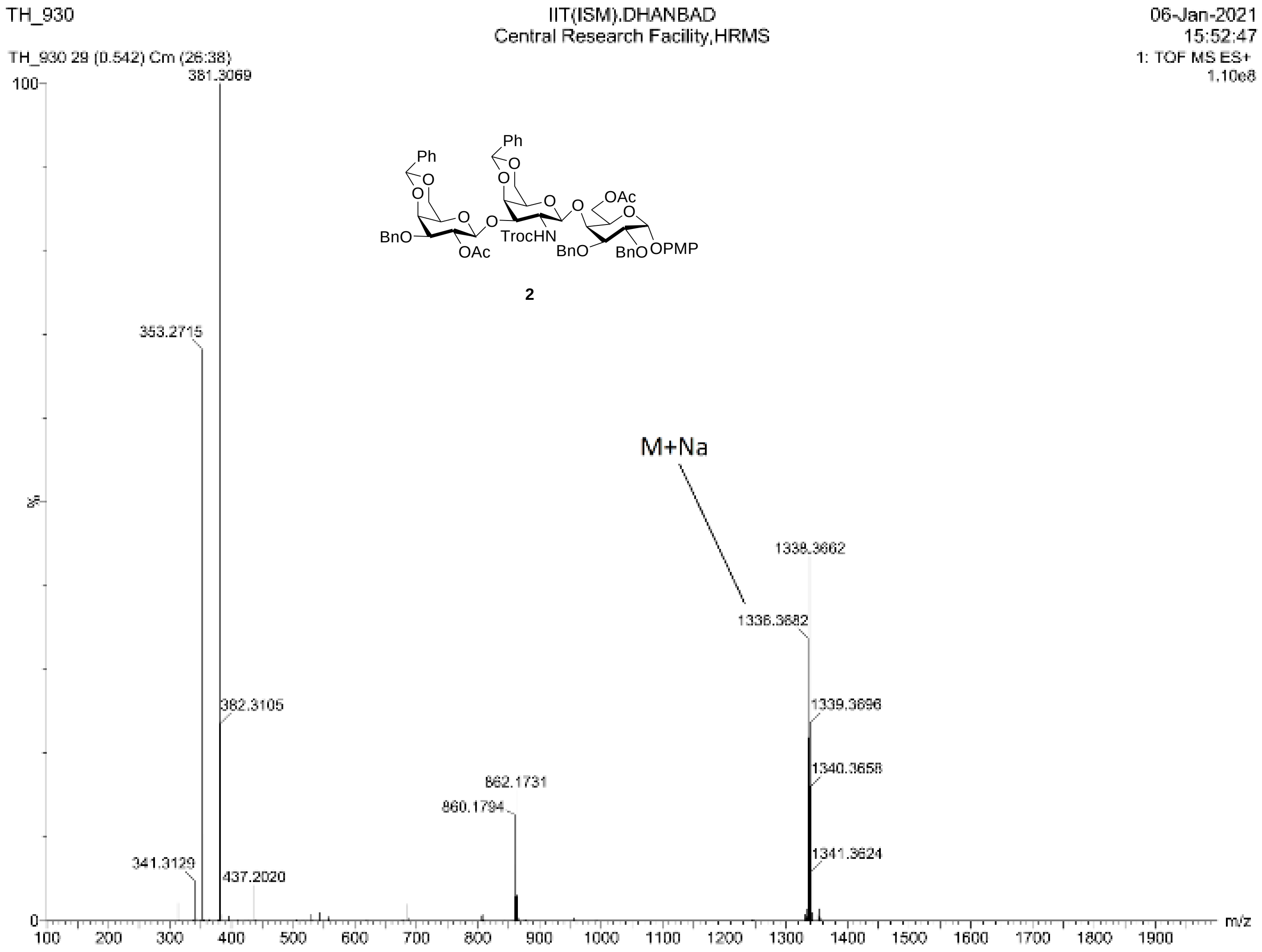

Figure S38: HRMS spectrum of compound **2** obtained from the one pot reaction.

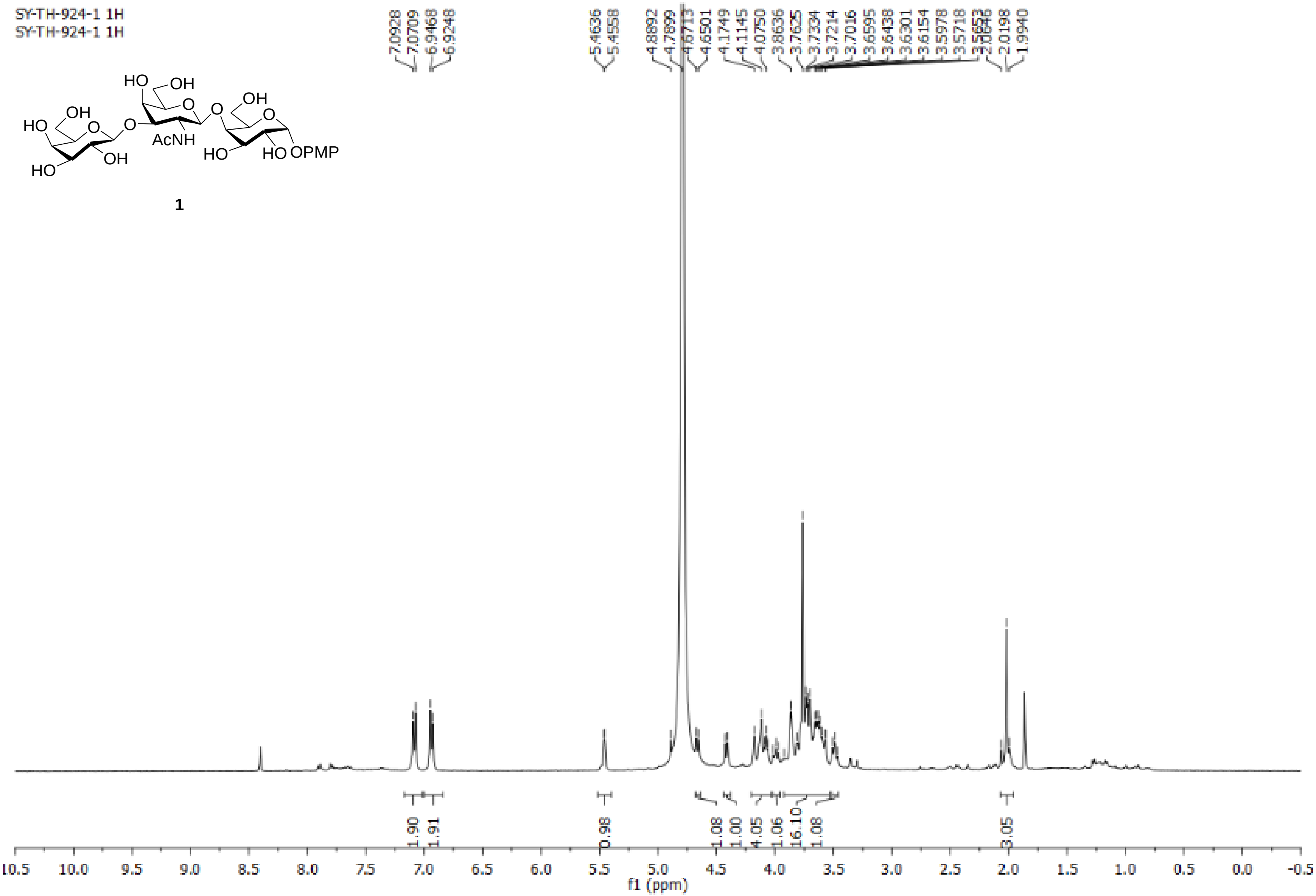

Figure S39: ^{1}H NMR spectrum (400 MHz, D_2O) of compound **1**.

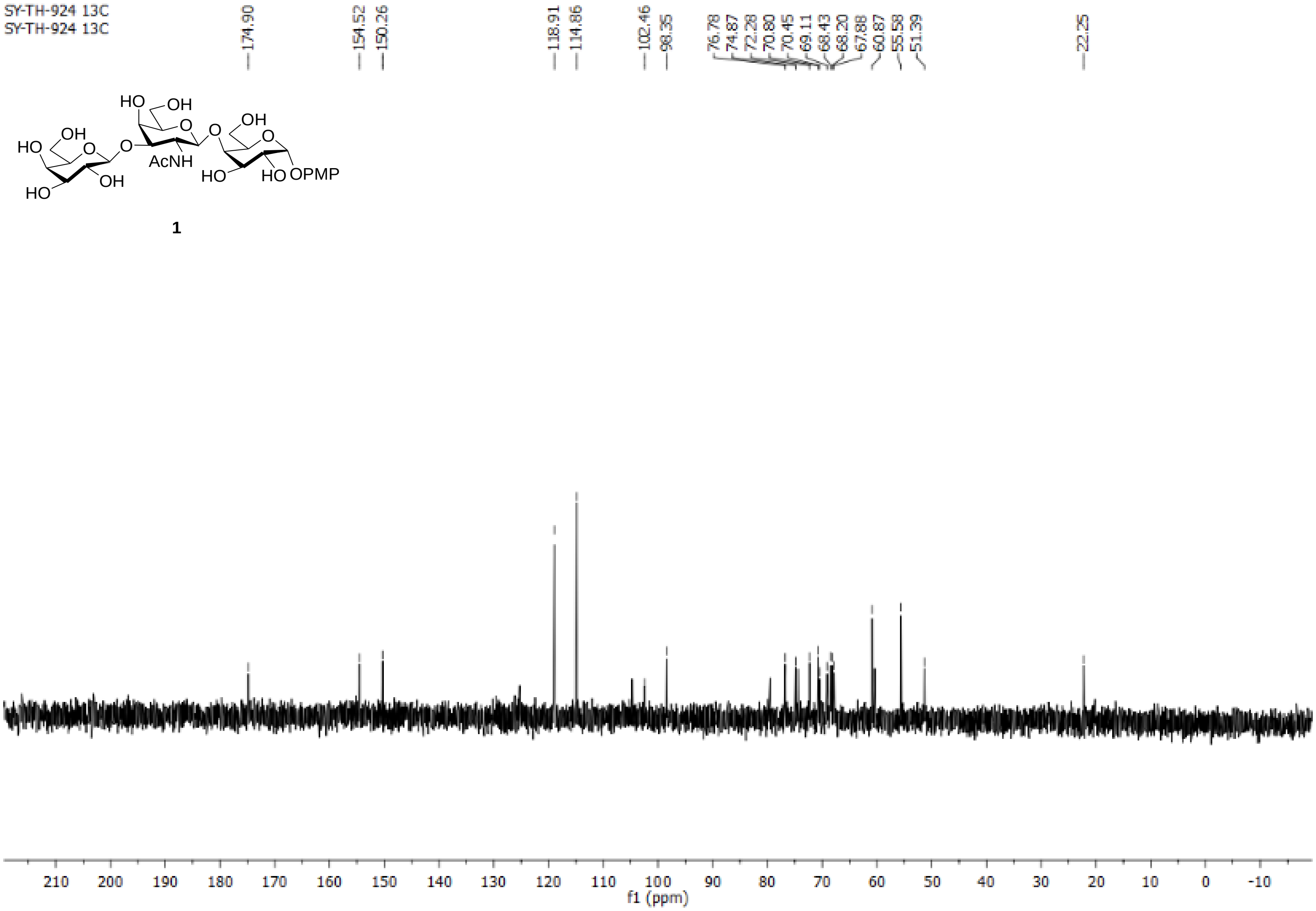

1

Figure S40: ^{13}C NMR spectrum (100 MHz, D_2O) of compound **1**.

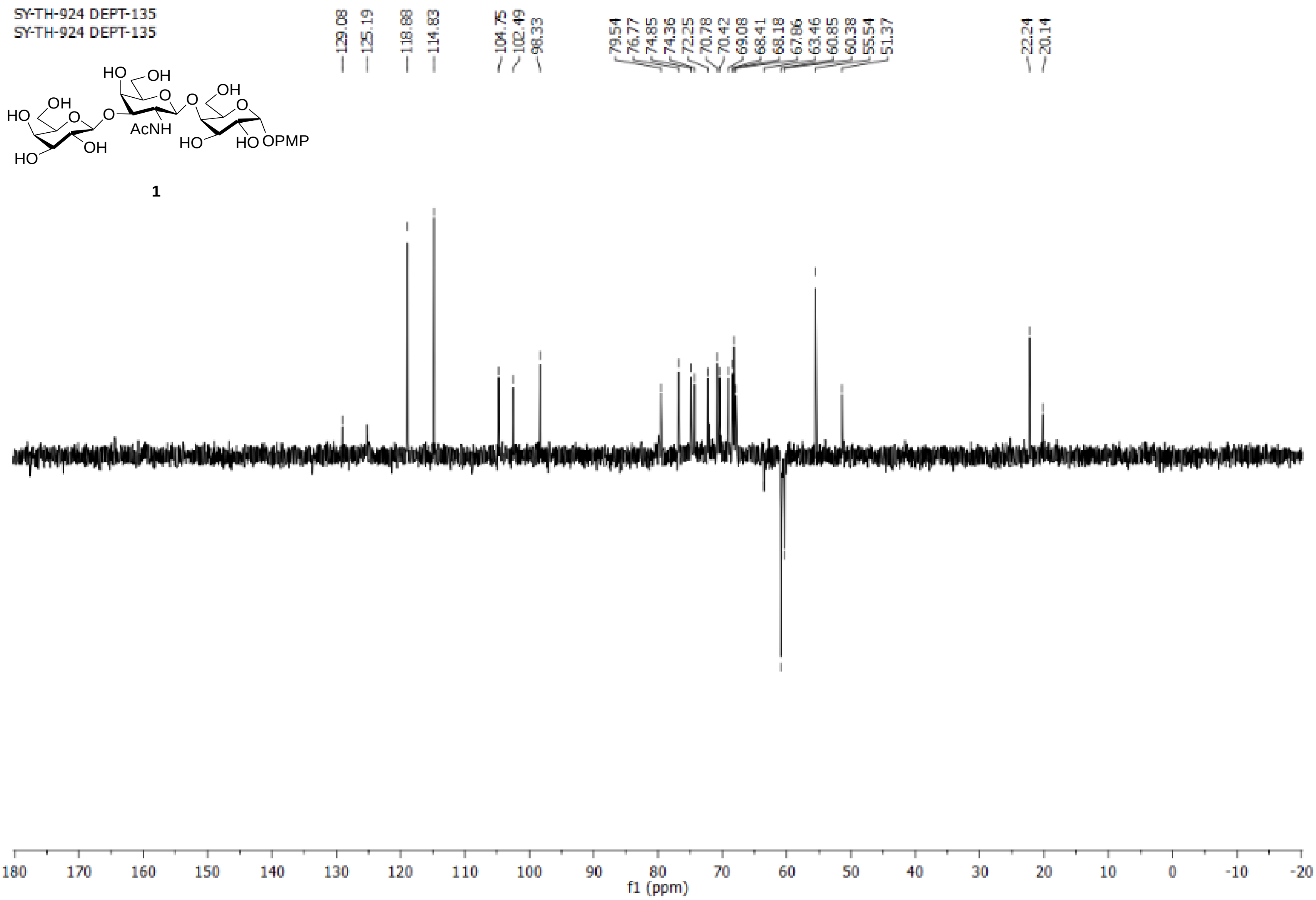

Figure S41: DEPT-135-NMR spectrum (100 MHz, D_2O) of compound **1**.

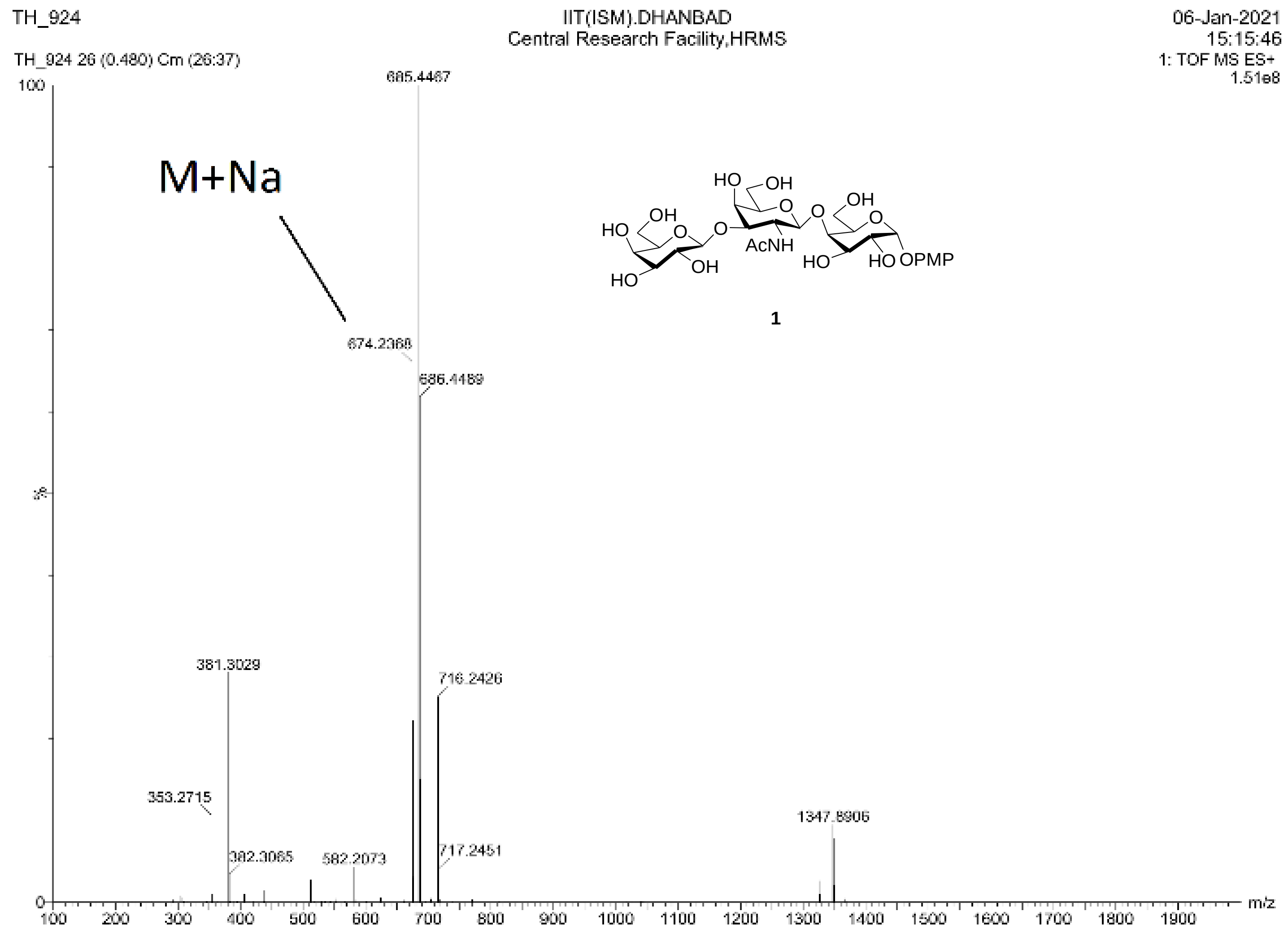

Figure S42: HRMS spectrum of compound **2**.
